# Hard- and soft-tissue changes after adult Class II Division 1 orthodontic treatment: a retrospective CBCT-based observational study

**DOI:** 10.3389/fmed.2026.1911048

**Published:** 2026-08-06

**Authors:** Zhenqi Zhao, Yun Gu, Xiaorong Tang, Pengcheng Gao

**Affiliations:** 1 Department of Stomatology, Nantong First People's Hospital, Nantong, Jiangsu, China; 2 Department of Stomatology, Zhongda Hospital, Southeast University, Nanjing, Jiangsu, China

**Keywords:** adult, Angle Class II Division 1 malocclusion, cephalometry, cone-beam computed tomography, measurement error, orthodontic treatment, soft tissue, stability

## Abstract

**Background:**

Restoring facial balance is now central to adult orthodontic care, yet evidence describing hard-soft tissue coupling and short-term retention stability in non-growing adults with Angle Class II Division 1 malocclusion remains limited.

**Methods:**

In this observational study, 236 consecutively treated adults (≥18 years) underwent clinically archived cone-beam computed tomography (CBCT) before treatment (T0) and within 2 weeks after debonding (T1). Ten hard- and five soft-tissue variables were measured on the T0 and T1 three-dimensional reconstructions and used for the treatment-change analysis. For the 12-month analysis (T2, *n* = 224) the T0 and T1 volumes were reformatted into orthogonally synthesized lateral cephalograms and re-measured, so that stability was assessed between two lateral cephalograms of the same geometrical type, the T2 record being the standardized digital lateral cephalogram taken at the routine retention review. Each T1–T2 change was judged against a variable-specific combined smallest detectable difference (SDD) derived from the method error of both cephalometric records, at group and at individual level. Paired tests with mean change, 95% *CIs* and Cohen's *dz*, correlations with multiplicity control, change-score correlations and multivariable linear regression evaluated change and hard–soft coupling.

**Results:**

From T0 to T1, ANB, U1-NA and L1-NB decreased and the interincisal angle increased (all *P* < 0.001), while SNA and SNB were unchanged. The nasolabial and mentolabial angles and chin thickness increased, and both lips retracted toward the aesthetic plane (all *P* < 0.001). Between T1 and T2 no variable showed a mean change that reached its combined SDD, and 92.5% to 98.7% of the T0–T1 change was still present at 1 year; 2.2%−6.7% of individual patients nevertheless crossed the SDD in the relapse direction. In adjusted models, maxillary incisor retraction remained independently associated with nasolabial-angle increase (*B* = −0.96° per degree; 95% *CI* −1.20 to −0.72) and with upper-lip retraction (*B* = +0.24 mm per millimeter; 95% *CI* +0.18 to +0.30), and mandibular incisor retraction with lower-lip retraction (*B* = +0.16; 95% *CI* +0.10 to +0.22). Adjusted *R*^2^ ranged from 0.146 to 0.397 and the maximum variance inflation factor (VIF) was 1.76.

**Conclusions:**

In non-growing adults, treatment was accompanied by predominantly dentoalveolar correction and by coupled soft-tissue change that was maintained at 1 year at group level, no mean change exceeding the error of the method. The coupling was consistent on average but explained a minority of the between-patient variance, and a small proportion of individuals showed measurable rebound, so the individual soft-tissue response remains difficult to predict. These observational data describe association and short-term stability, not causal benefit.

## Introduction

Malocclusion is among the most prevalent oral conditions worldwide, and Angle Class II Division 1 is one of its most frequently encountered forms (1, 2). It is characterized by a distal molar relationship, proclined and protrusive maxillary incisors, increased overjet, a convex profile, and lip incompetence; the prominent incisors are also disproportionately exposed to trauma and may carry psychosocial consequences (3, 4). Its etiology is multifactorial, reflecting genetic and environmental influences on the dentition, the skeletal bases, and the perioral musculature.

Over the past two decades the goals of treatment have broadened from occlusal correction to the optimization of facial balance, and adults increasingly seek care for the appearance of the lips and profile (5, 6). In daily practice, adult patients frequently ask not only whether incisor retraction will improve lip protrusion, but also how much change is realistic and whether it will remain stable after appliance removal. Because the overlying soft tissues do not move in fixed proportion to the teeth and bone, the soft-tissue response to incisor retraction is variable and only partially predictable, and it has therefore been studied extensively (7, 8). Reported ratios of upper-lip to maxillary-incisor retraction span roughly 1:0.2–1:0.9 across published series, and lip thickness, lip strain and perioral muscle tone each modify the response, so that a reassuring group mean can conceal a wide spread of individual outcomes (9, 10). Two careful series that referenced incisor movement to miniscrews or to a rough-surfaced palatal implant found that much of the variance remained unexplained even when the dental movement itself was measured with negligible error (11, 12). Quantifying this response in adults is clinically important because growth-related skeletal adaptation is minimal, making treatment-related dentoalveolar and soft-tissue relationships easier to interpret.

Accurate appraisal depends on imaging and standardized measurement. Two-dimensional cephalometry remains widely used but is limited by projection error, magnification, head-position variation and superimposition of bilateral structures. Cone-beam computed tomography (CBCT) reconstructs craniofacial anatomy in three dimensions, supporting more reproducible landmark identification and individualized planning (13–16). At the same time, CBCT involves ionizing radiation; therefore, retrospective analyses should rely on clinically available imaging and avoid additional research-only exposure whenever possible. A three-dimensional volume can, however, be reformatted into an orthogonally synthesized lateral cephalogram, which makes it possible to compare an archived CBCT record with a conventional two-dimensional record acquired later in the same patient without exposing that patient again.

Most evidence on the soft-tissue effects of treatment, however, derives from adolescent or mixed-age samples in whom residual growth confounds interpretation, and few studies have quantified hard- and soft-tissue change simultaneously, and exclusively, in non-growing adults using three-dimensional imaging (6, 17, 18). Moreover, many studies report associations between final hard- and soft-tissue positions, but final-state correlations do not necessarily show that the magnitude of dental movement explains the magnitude of soft-tissue change. Two questions are therefore especially relevant for adult care and remain incompletely answered: whether the amount of soft-tissue change is coupled to the amount of hard-tissue change, and whether the changes observed at debonding are retained during the first year of retention.

We therefore characterized hard- and soft-tissue change from before treatment to debonding, its coupling through change-score and adjusted analyses, and its 12-month clinical stability, in adults with Angle Class II Division 1 malocclusion. We pre-specified that (i) treatment would be accompanied by measurable soft-tissue profile change, namely an increased nasolabial angle and mentolabial sulcus angle, increased soft-tissue chin thickness, and reduced UL–E and LL–E distances; (ii) the magnitude of dentoalveolar change (ΔANB, ΔU1-NA, ΔL1-NB, ΔU1-L1) would correlate with the magnitude of soft-tissue change; and (iii) the observed changes would be largely retained at 12 months. A fourth aim was added during analysis: to report how much of the between-patient variance in soft-tissue change is actually explained by the dentoalveolar change, and to judge 1-year stability against the measurement error of the records from which it is estimated rather than against a percentage. The study characterizes change, coupling and short-term stability rather than establishing causation; analyses are descriptive, correlational and association-based.

## Materials and methods

### Study design and setting

**Figure 1:**
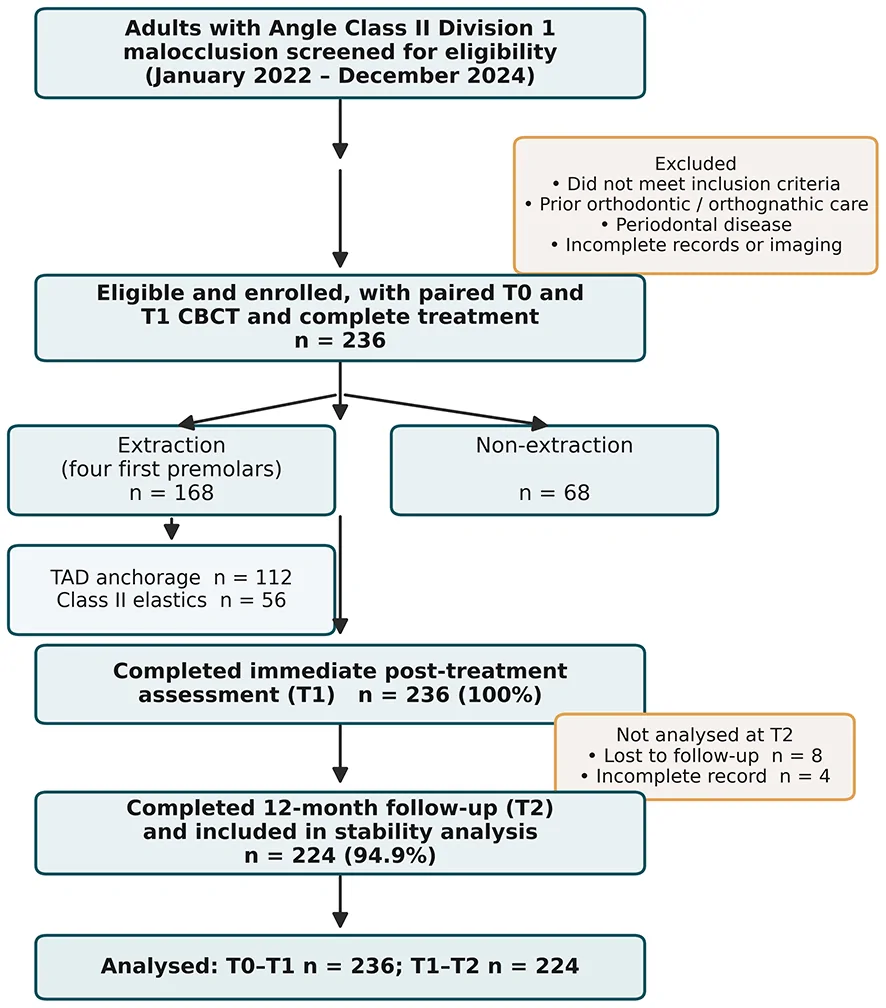
Participant flow through screening, treatment, and 12-month follow-up (STROBE).

This observational study, comprising a cone-beam computed tomography (CBCT) based pre-treatment and post-treatment analysis together with a 12-month follow-up assessment, was conducted at the Department of Stomatology, Nantong First People's Hospital, which recruited, treated and followed all patients and which holds the clinical records and acted as data custodian. The Department of Stomatology, Zhongda Hospital, Southeast University, contributed to the design of the analysis, to its supervision and to the interpretation of the results. The protocol, including the retrospective use of clinically archived CBCT volumes, lateral cephalograms and treatment records, was reviewed and approved by the Ethics Committee of Nantong First People's Hospital, Nantong, Jiangsu, China (approval number 2024KT002; approved February 01, 2024), which is the ethics committee of record for the institution at which the patients were treated and in which the imaging and treatment records are held. The study was conducted in accordance with the Declaration of Helsinki and its later amendments. All patients had given written consent for the use of their anonymized clinical and imaging data for research at the time of treatment; no imaging or other procedure was performed for the purposes of this study, and no identifiable information is presented. Reporting follows the Strengthening the Reporting of Observational Studies in Epidemiology (STROBE) guidance (19), and the participant pathway is shown in Figure 1.

### Participants

Adults with Angle Class II Division 1 malocclusion who completed comprehensive fixed-appliance treatment between January 2022 and December 2024 were consecutively screened. Inclusion criteria were: age ≥18 years; mild anterior crowding ( ≤ 4 mm) with overjet >4 mm; a bilateral distal (Class II) molar relationship at maximum intercuspation; fully erupted canines without marked rotation or ectopia, intact crowns without severe wear, and complete root formation; and a complete permanent dentition (with or without third molars) without supernumerary teeth or spacing. Exclusion criteria were a history of cleft lip or palate, maxillofacial trauma or surgery, jaw pathology, or congenital syndrome; previous orthodontic or orthognathic treatment; congenital absence of teeth; and active periodontal disease.

### CBCT acquisition and radiation safety

CBCT examinations at T0 and T1 were retrieved from the institutional clinical imaging archive. All scans were acquired according to departmental imaging protocols using standardized acquisition parameters and radiation-minimization principles. No additional imaging was requested by the investigators for the purposes of this analysis. All CBCT scans were acquired by one technician on the same unit (KaVo 3D eXam i; KaVo Dental, Germany) using an identical mode [120 kV, 5 mA, 23-s scan time, 0.2-mm isotropic voxel, and a 16 × 13 cm field of view extending from the cranial base to the soft-tissue menton and encompassing the whole facial profile; Digital Imaging and Communications in Medicine (DICOM) export]. Participants were seated with the Frankfort plane parallel to the floor and the mid-sagittal plane perpendicular to it. Lip posture was actively standardized rather than assumed: each patient was asked to swallow, to bring the teeth gently into maximum intercuspation, and then to let the lips rest in light contact without conscious effort, in the relaxed repose position (20). Head position was reproduced by the self-balance method, the patient looking into a mirror at eye level, a procedure whose reproducibility has been quantified (21). The same verbal instruction was used at every time point. Scans showing clinically significant motion, or a head or lip posture that departed from this standard, were regarded as unsuitable for measurement and were excluded under the departmental imaging-quality criteria; no scan was repeated for research purposes. The forehead and chin supports were used only for positional stabilization; care was taken to avoid contact with or compression of the soft-tissue pogonion and labiomental region used for measurement. The 12-month follow-up assessment (T2) required no additional research-only imaging; the record used and the steps taken to make it comparable with T1 are described in the next subsection.

### Twelve-months follow-up record and modality-matched measurement

Every patient in this department attends a retention review 12 months after debonding, at which a digital lateral cephalogram is acquired as part of routine care. That radiograph, and no other record, is the source of every T2 value reported here; photographs and study casts were inspected only to confirm retainer wear and the absence of occlusal settling and contributed no measurement. All films were taken by the same radiographer on a single cephalostat with ear rods, with the Frankfort plane parallel to the floor, the teeth in maximum intercuspation and the lips in relaxed repose, using the instruction script described above. Source-to-image and source-to-object distances were fixed, so that magnification was constant and was removed by calibration against the ruler embedded in the cephalostat.

A three-dimensional measurement and a two-dimensional measurement of nominally the same variable are not the same quantity, and a difference between them therefore confounds biological change with a change of measurement geometry. The stability analysis was consequently not performed on the three-dimensional T1 values. Instead, each T0 and T1 volume was reformatted by ray-sum projection, at the nominal geometry of the cephalostat, into an orthogonally synthesized lateral cephalogram, and the 15 variables defined in Supplementary Table S1 were re-measured on those projections. Cephalograms synthesized in this way have been validated against conventional films in dry skulls and in patients, with discrepancies for standard cephalometric variables of the order of one degree and half a millimeter (22–24). The T1–T2 contrast reported in this paper is therefore between two lateral cephalograms of the same geometrical type. The three-dimensional volumes are retained, unchanged, for the T0–T1 treatment-change analysis, which is where their advantage lies. Three-dimensional and cephalometric values were held in separately named variables throughout and were never interchanged.

Reliability was established separately for each image type, and the resulting method errors and combined smallest detectable differences are given in Supplementary Table S2. The cost of the projection was measured rather than assumed. In 40 randomly selected patients the same T1 scan was measured twice, once volumetrically and once on its synthesized projection, so that any difference is attributable to the projection alone. Mean bias, 95% limits of agreement and intraclass correlation coefficients (ICC) for all 15 variables, together with a regression of the difference on the average to test for proportional bias, are reported in Supplementary Table S3.

### Orthodontic treatment

All participants received comprehensive fixed-appliance treatment with pre-adjusted edgewise brackets (0.022-inch slot, Roth prescription). Treatment decisions were made before appliance placement by the treating orthodontist together with the supervising consultant, for clinical reasons and not for the purposes of this analysis; allocation was not randomized. Extraction of four first premolars was chosen when at least two of the following were present: overjet greater than 6 mm; a protrusive lip profile, defined as the upper lip lying more than 2 mm anterior to the aesthetic plane; a maxillary arch-length discrepancy of at least 4 mm; and a planned incisor retraction that could not be achieved by expansion or distalisation without proclining the mandibular incisors beyond 95° to the mandibular plane. On this basis 168 patients (71.2%) underwent extraction and 68 (28.8%) were treated without extraction. Anchorage was chosen by a separate rule: temporary anchorage devices (TADs) were used when more than two thirds of the extraction space was to be taken by anterior retraction (*n* = 112), and Class II intermaxillary elastics were used when moderate anchorage was sufficient (*n* = 56). Treatment followed a standard sequence of leveling and alignment (0.014- to 0.018-inch nickel–titanium archwires), space closure, finishing and retention, and space closure in every extraction case used sliding mechanics on a 0.019 × 0.025-inch stainless-steel archwire, so that archwire stiffness during retraction was constant across the cohort.

TADs were self-drilling miniscrews of 1.6 mm diameter and 8 mm length, placed under local anesthesia in the buccal alveolar bone between the maxillary second premolar and first molar, 8–10 mm apical to the alveolar crest and clear of the roots. Two maxillary screws were placed in every TAD patient, and two mandibular screws were added in 18 of them. Retraction force was applied from the miniscrew head to a soldered hook on the archwire between the lateral incisor and the canine by a nickel–titanium closed coil spring delivering approximately 150 g per side, reactivated at each four-weekly visit. Class II elastics, where used, were 3/16 inch and 4.5 ounces, worn full time except during meals and tooth-brushing and changed daily, fitted from the maxillary canine hook to the mandibular first molar hook and begun only after the mandibular arch had been leveled on a rectangular stainless-steel wire; adherence was recorded at each visit from the patient diary and from elastic consumption.

Retention was also assigned by written indication. Every patient received a maxillary vacuum-formed retainer. In the mandibular arch, a bonded canine-to-canine fixed retainer was placed when any of the following applied: a pre-treatment Little irregularity index greater than 3 mm, treatment involving extraction, or derotation of a mandibular incisor by more than 20°. A vacuum-formed retainer alone was used when none applied, and a combined fixed and removable regimen when a fixed retainer was indicated and the overbite had been increased during treatment. Retainers were worn full time for 6 months and at night thereafter, and adherence was recorded at every review from the patient diary and from inspection of the appliance.

### Cephalometric measurements

The DICOM datasets were imported into eXamVision software, version 1.9.3.13 (KaVo Dental GmbH, Biberach, Germany). Each volume was oriented before any landmark was placed: the axial plane was set parallel to the Frankfort horizontal defined by the right and left porion and the right orbitale, the coronal plane perpendicular to it through both porion points, and the mid-sagittal plane through nasion, basion and anterior nasal spine, with rotation corrected in all three planes. Landmarks were then identified on the multiplanar reconstruction rather than on a rendered surface, each landmark being confirmed in at least two orthogonal views before it was accepted; soft-tissue landmarks were identified on the volume rendering with the threshold fixed at the value used for every patient, so that vermilion boundaries were not operator-dependent. Ten hard-tissue variables (SNA, SNB, ANB, the *Y*-axis angle, U1-NA angular and linear, L1-NB angular and linear, and U1-L1 angular and linear) and five soft-tissue variables (the nasolabial angle, the mentolabial sulcus dip angle, the UL–E and LL–E line distances to the Ricketts aesthetic plane, and soft-tissue chin thickness, defined as the distance between hard-tissue and soft-tissue pogonion) were recorded. The same landmark and plane definitions were applied, without modification, to the synthesized T1 cephalograms and to the conventional T2 cephalograms. All 15 variables were measurable on the 12-month cephalogram in every one of the 224 patients retained in the stability analysis; no value was imputed and no variable-specific exclusion occurred. The U1-L1 linear measurement was defined as the shortest distance between the incisal edges of the maxillary and mandibular central incisors on the mid-sagittal reconstruction. All landmark and plane definitions are given in Supplementary Table S1.

### Measurement reliability

All landmarks were identified and all variables measured by a single trained examiner blinded to time point and treatment allocation. A random subset of 30 scans was re-measured by the same examiner after a 2-week interval, and independently by a second examiner, to assess intra- and inter-examiner reliability with the intraclass correlation coefficient (ICC; two-way mixed-effects model, absolute agreement); systematic error was tested with paired *t*-tests and random error with Dahlberg's formula. Because reliability established on a three-dimensional volume does not transfer to a projection, the same procedure was repeated in 30 randomly selected records of each type for the synthesized T1 cephalograms and for the conventional T2 cephalograms. On the three-dimensional reconstructions, intra-examiner ICCs ranged from 0.958 to 0.988 and inter-examiner ICCs from 0.931 to 0.972, and the Dahlberg error was no greater than 0.89° for angular and 0.22 mm for linear variables. On the conventional T2 cephalograms, intra-examiner ICCs ranged from 0.951 to 0.982 and inter-examiner ICCs from 0.921 to 0.964, and the Dahlberg error was no greater than 1.02° and 0.25 mm. No systematic error was detected in any comparison (all *P* > 0.05). Because the stability contrast involves one synthesized and one conventional cephalogram, the measurement error that matters is that of the two two-dimensional records taken together. For each variable a combined smallest detectable difference was therefore computed as *SDD* = 1.96 × √(DE^2^T1 + DE^2^T2), where DE is the Dahlberg error of the record concerned. This is the smallest change in an individual that can be separated from method error with 95% confidence (25, 26). Variable-level ICCs with confidence intervals, method errors and combined SDD values are reported in Supplementary Table S2.

### Sample size

Because all consecutively eligible patients treated during a fixed window were analyzed, no *a priori* calculation was undertaken. With 236 paired observations the study afforded >99% power (α = 0.05, two-sided) to detect a standardized paired difference of 0.30 and ≈80% power to detect a correlation of |*r*| ≥ 0.18.

### Statistical analysis

Analyses used SPSS 26.0 (IBM Corp., Armonk, NY, USA). Normality of paired differences and of raw values was confirmed (Shapiro–Wilk, Q–Q plots). The treatment-change analysis uses the three-dimensional dataset alone. For each variable we report the mean at T0 and T1, the T0–T1 mean change with its 95% confidence interval, the paired *t*-test and Cohen's *dz*. The stability analysis uses the two-dimensional dataset alone. For each variable we report the T0–T1 change measured on the synthesized cephalograms, the paired T1–T2 mean change with its 95% confidence interval, the combined SDD, the number and proportion of individual patients whose T1–T2 change exceeded the SDD in the relapse direction, and, for continuity with the earlier literature, the percentage of the T0–T1 change still present at 12 months. A group mean below the SDD indicates that the average change cannot be distinguished from method error; it does not establish equivalence, and it does not describe the individual patient, which is why the individual-level exceedance is reported alongside it. This measurement-error criterion replaced the qualitative stability categories used in the original analysis plan and should be regarded as a revised rather than a pre-specified criterion. Post-treatment hard–soft associations were examined at T1 and, when corresponding T2 data were available, at 12 months with Pearson correlations and 95% *CIs*; because 25 correlations were tested, the false-discovery rate was controlled (Benjamini–Hochberg) and associations surviving a Bonferroni threshold (α = 0.05/25 = 0.002) are identified. Spearman correlations were computed as a sensitivity analysis. To address the clinically central question of whether the magnitude of hard-tissue change explains the magnitude of soft-tissue change, change-score (Δ-Δ) correlations were calculated, and multivariable linear regression modeled Δ nasolabial angle, ΔUL–E, ΔLL–E, Δ mentolabial angle and Δ chin thickness on the relevant hard-tissue change while adjusting for age, sex, the baseline value of the outcome, extraction status and treatment duration. Anchorage was deliberately excluded from the full-cohort models: because every TAD patient and every elastics patient was an extraction patient, extraction status is the exact sum of the two anchorage indicators, and entering all three would have produced perfect linear dependence. Anchorage was therefore examined only within the 168 extraction patients, in otherwise identical models comparing TADs with Class II elastics. Coefficients are reported as unstandardized *B* with 95% confidence intervals, together with the standardized β, so that the two are not confused. Before interpretation every model was screened for linearity and homoscedasticity (standardized residuals against fitted values; Breusch–Pagan test), normality of residuals (normal probability plot; Shapiro–Wilk test), independence (Durbin–Watson statistic), influence (Cook's distance with a 4/n threshold, and leverage) and multicollinearity (tolerance and variance inflation factor, VIF), a VIF above 5 being taken as the threshold for concern while recognizing that such thresholds are conventions rather than tests (27). Model fit is reported as *R*^2^ and adjusted *R*^2^ with the overall *F* statistic. Subgroup and sensitivity analyses compared extraction with non-extraction patients, TADs with Class II elastics among extraction cases, and the three retention regimens, and re-estimated the principal models with retainer type added as a covariate. The 12-month analysis was complete-case (*n* = 224); baseline characteristics of patients with and without follow-up were compared using standardized mean differences, a value below 0.20 being taken to indicate no meaningful imbalance (28). Because treatment was allocated by clinical indication, no attempt was made to quantify residual confounding; it is discussed qualitatively instead. A two-sided *P* < 0.05 was significant. All correlations are descriptive and do not imply causation.

## Results

### Participants and baseline characteristics

Of the consecutively screened patients, 236 met all criteria and completed both T0 and T1 CBCT assessment and the full treatment protocol (Figure 1); 224 (94.9%) had available 12-month (T2) clinical follow-up records. The cohort comprised 100 men and 136 women, with a mean age of 21.4 ± 3.1 years. Baseline and treatment characteristics for the whole cohort and for the extraction and non-extraction groups are given in Table 1, and the treatment mechanics, retention regimens and adherence are tabulated in Supplementary Table S4. Extraction patients had, as expected, slightly larger baseline overjet and crowding and longer treatment duration, reflecting the greater dentoalveolar correction required in this subgroup. The 12 patients without a 12-month record (eight lost to follow-up, four with an incomplete record set) did not differ appreciably at baseline from the 224 retained: no standardized mean difference exceeded 0.20 for age, sex, overjet, crowding, ANB, U1-NA, UL–E, extraction status, anchorage or treatment duration (Supplementary Table S5). Selection bias arising from unmeasured differences cannot be excluded, but no observable imbalance supports the complete-case stability analysis.

**Table 1:** Baseline and treatment characteristics.

Characteristic	Overall (*n* = 236)	Extraction (*n* = 168)	Non-extraction (*n* = 68)
Age, years	21.4 ± 3.1	21.6 ± 3.2	20.9 ± 2.8
Female, *n* (%)	136 (57.6)	99 (58.9)	37 (54.4)
Male, *n* (%)	100 (42.4)	69 (41.1)	31 (45.6)
Baseline overjet, mm	6.8 ± 1.1	7.1 ± 1.0	6.1 ± 0.8
Baseline crowding, mm	2.7 ± 0.9	3.1 ± 0.8	1.8 ± 0.7
Treatment duration, months	21.3 ± 4.2	22.2 ± 4.0	19.1 ± 3.8
Four first-premolar extraction, *n* (%)	168 (71.2)	168 (100)	0
Temporary anchorage devices, *n* (%)	112 (47.5)	112 (66.7)	0
Class II elastics, *n* (%)	56 (23.7)	56 (33.3)	0
Full-time retainer for 6 months, *n* (%)	221 (93.6)	157 (93.5)	64 (94.1)
Twelve-month follow-up available, *n* (%)	224 (94.9)	158 (94.0)	66 (97.1)

Values are mean ± SD or n (%).

### Hard- and soft-tissue change from T0 to T1

**Figure 2:**
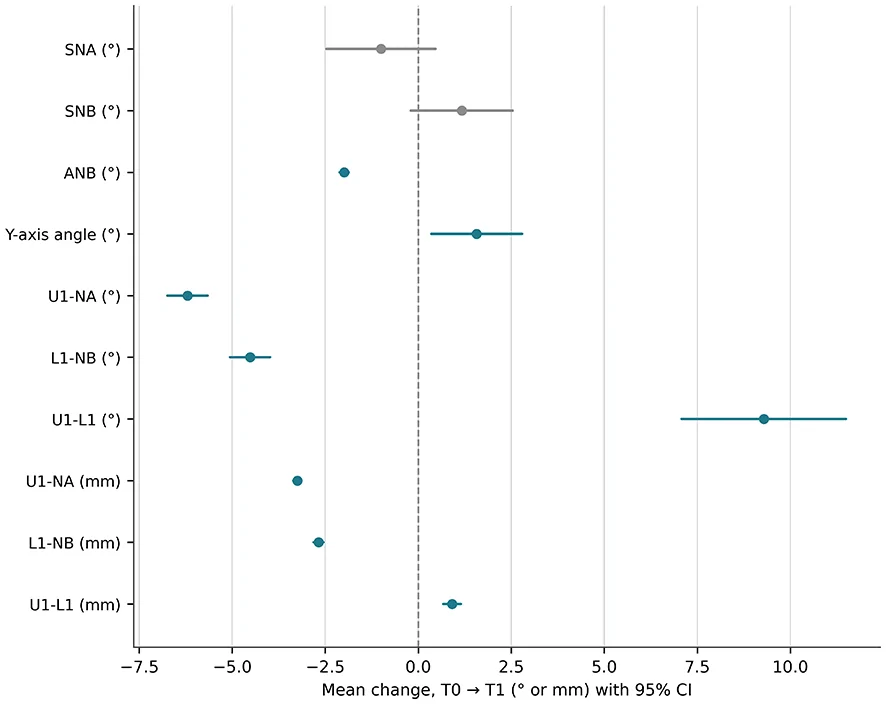
Hard-tissue variables. Mean change from T0 to T1 with its 95% confidence interval, measured on the three-dimensional CBCT reconstructions (*n* = 236). Teal indicates a statistically significant change; gray indicates a change that was not significant. Twelve-month stability is shown separately, in Figure 4, because it is estimated from a different type of image.

**Figure 3:**
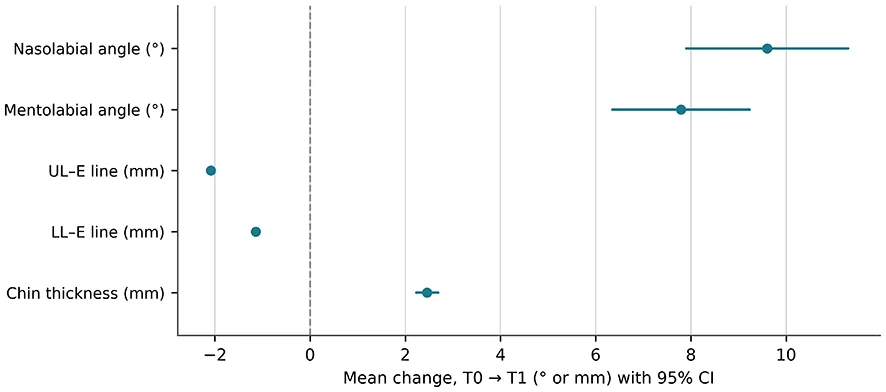
Soft-tissue variables, presented as in Figure 2: mean change from T0 to T1 with its 95% confidence interval, measured on the three-dimensional CBCT reconstructions (*n* = 236).

From T0 to T1, SNA and SNB were unchanged (*P* = 0.181 and 0.092), consistent with skeletal stability in this non-growing cohort. In contrast, ANB, U1-NA and L1-NB (angular and linear) decreased, while the *Y*-axis angle and U1-L1 (angular and linear) increased (all *P* ≤ 0.011), indicating that correction was concentrated at the dentoalveolar level rather than in the basal skeletal relationship. The largest standardized changes were observed for incisor prominence and lip position. The nasolabial and mentolabial angles and soft-tissue chin thickness increased, whereas the UL–E and LL–E distances decreased (all *P* < 0.001), indicating reduced lip protrusion relative to the aesthetic plane. Means, mean changes with 95% CIs, Cohen's *dz* and *P*-values are presented in Table 2 and in Figures 2, 3. All values in this section derive from the three-dimensional reconstructions; no two-dimensional measurement enters the treatment-change analysis.

**Table 2:** Hard- and soft-tissue measurements at T0 (pre-treatment) and T1 (debonding), with the T0–T1 mean change, its 95% *CI*, Cohen's *dz* and the paired *t*-test *P*-value (*n* = 236).

Variable	T0	T1	Change T0–T1 (95% CI)	*dz*	*P*
Hard tissue					
SNA (°)	81.26 ± 8.18	80.26 ± 8.05	−1.00 (−2.47, 0.47)	0.09	0.181
SNB (°)	75.11 ± 7.46	76.28 ± 7.63	+1.17 (−0.20, 2.54)	0.11	0.092
ANB (°)	7.35 ± 0.74	5.36 ± 0.54	−1.99 (−2.11, −1.87)	2.17	< 0.001
*Y*-axis angle (°)	66.45 ± 6.65	68.02 ± 6.82	+1.57 (0.35, 2.79)	0.17	0.011
U1-NA (°)	32.28 ± 3.26	26.08 ± 2.62	−6.20 (−6.74, −5.66)	1.48	< 0.001
L1-NB (°)	31.76 ± 3.18	27.24 ± 2.73	−4.52 (−5.06, −3.98)	1.08	< 0.001
U1-L1 (°)	117.25 ± 11.68	126.54 ± 12.68	+9.29 (7.08, 11.50)	0.54	< 0.001
U1-NA (mm)	7.30 ± 0.73	4.05 ± 0.41	−3.25 (−3.36, −3.14)	3.88	< 0.001
L1-NB (mm)	8.08 ± 0.83	5.40 ± 0.54	−2.68 (−2.81, −2.55)	2.71	< 0.001
15.6-7.2,-1.3499ptU1-L1 (mm)	12.60 ± 1.27	13.51 ± 1.35	+0.91 (0.67, 1.15)	0.49	< 0.001
Soft tissue					
Nasolabial angle (°)	88.70 ± 8.87	98.30 ± 9.85	+9.60 (7.90, 11.30)	0.72	< 0.001
Mentolabial angle (°)	75.26 ± 7.53	83.05 ± 8.32	+7.79 (6.35, 9.23)	0.69	< 0.001
UL–E line (mm)	2.60 ± 0.26	0.52 ± 0.05	−2.08 (−2.11, −2.05)	7.86	< 0.001
LL–E line (mm)	3.56 ± 0.36	2.42 ± 0.25	−1.14 (−1.20, −1.08)	2.60	< 0.001
Soft-tissue chin thickness (mm)	11.08 ± 1.12	13.54 ± 1.36	+2.46 (2.23, 2.69)	1.40	< 0.001

All values were measured on the three-dimensional CBCT reconstruction. The 12-month follow-up measurements are reported separately, in Table 3, because they were made on lateral cephalograms and are not directly comparable with three-dimensional values; mixing the two geometries within one longitudinal series would be misleading.

Cohen's *dz* is the mean change divided by the standard deviation of the paired differences. The very large values for U1-NA (mm) and UL–E arise from the small dispersion of those paired differences in this cohort rather than from an unusually large treatment effect, and the same narrow range attenuates the intraclass correlation for UL–E in Supplementary Table S3, where the Bland and Altman limits are the more informative statistic. The mean changes and their confidence intervals, not dz, are the quantities intended to transfer to other populations. Reliability and method error for each variable are given in Supplementary Table S2.

### Twelve-months stability

The stability analysis was performed entirely on lateral cephalograms, the T0 and T1 films being synthesized from the corresponding CBCT volumes and the T2 film acquired conventionally. Agreement between the volumetric and the synthesized measurement of the same T1 scan was close for every variable, the mean bias never exceeding 0.18° or 0.06 mm and the limits of agreement lying within the combined SDD in each case (Supplementary Table S3), so the change of image type is unlikely to drive the T1–T2 contrast.

**Figure 4:**
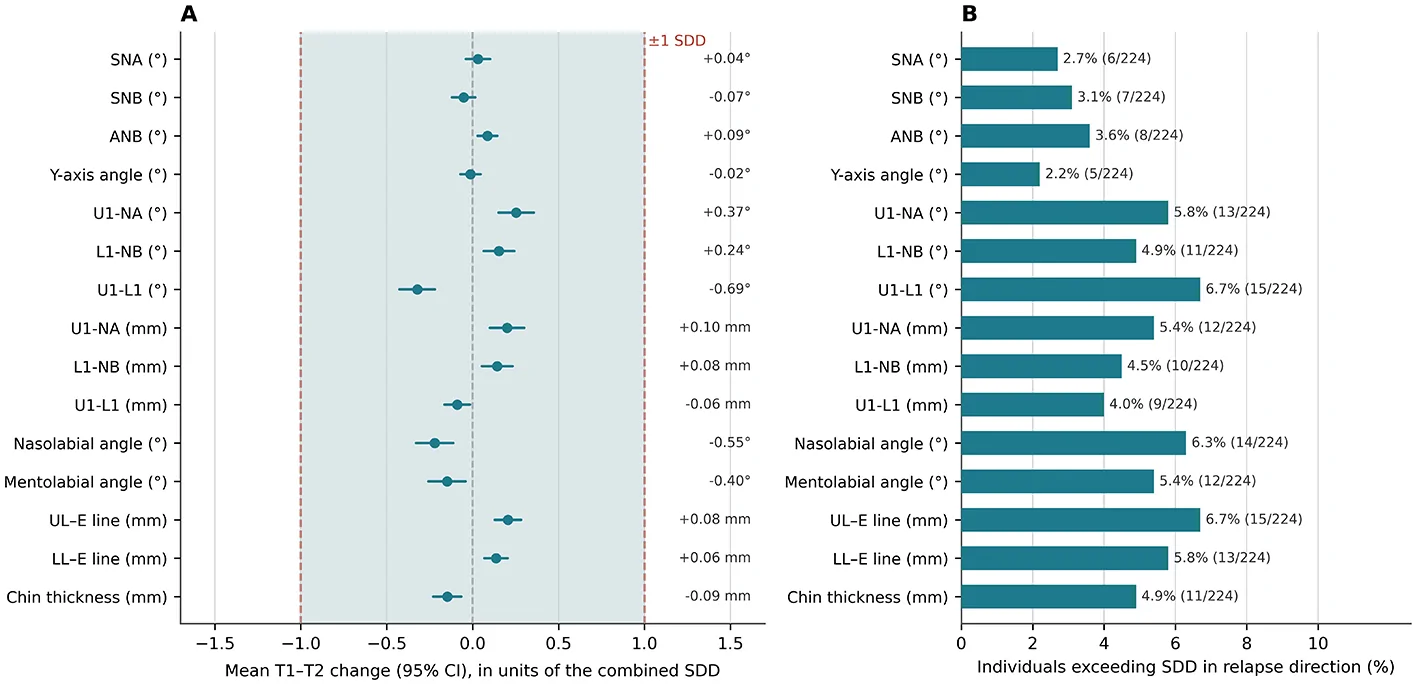
Twelve-month stability judged against measurement error, from the modality-matched cephalometric dataset (*n* = 224). **(A)** Mean T1–T2 change with its 95% confidence interval, expressed in units of the variable-specific combined smallest detectable difference (SDD = 1.96 × √(DE^2^T1 + DE^2^T2), where DE is the Dahlberg error of the record concerned). The shaded band spans ±1 SDD; a point and interval lying wholly inside it denotes a mean change that cannot be distinguished from the error of the method. Raw mean changes are annotated at the right of each row. **(B)** Percentage of individual patients whose T1–T2 change exceeded the SDD in the relapse direction, with the count shown beside each bar. Group-level stability **(A)**, coexists with measurable rebound in a small minority of individuals **(B)**.

Every mean T1–T2 change lay in the direction of the pre-treatment value, but each was small. The largest was 0.69° for the interincisal angle, against a combined SDD of 2.14° for that variable; the next largest were 0.55° for the nasolabial angle (SDD 2.49°) and 0.40° for the mentolabial angle (SDD 2.68°). No variable showed a mean change that reached its SDD, and for every variable the confidence limit furthest from zero also lay below the SDD; expressed as a fraction of the SDD, the mean changes ranged from 0.01 to 0.32 (Table 3 and Figure 4A). Between 92.5% (interincisal angle) and 98.7% (*Y*-axis angle) of the T0–T1 change was still present at 12 months. SNA and SNB, which had not changed appreciably during treatment, were likewise unchanged at follow-up.

**Table 3:** Twelve-months stability among complete cases (*n* = 224).

Variable	2D T0–T1 change	T1–T2 change (95% CI)	Combined SDD	|Mean| ÷SDD	Exceeding SDD in relapse direction	Change maintained (%)
SNA (°)	−0.92	+0.04 (−0.05, +0.13)	1.28	0.03	6/224 (2.7)	NA^*^
SNB (°)	+1.10	−0.07 (−0.16, +0.02)	1.33	0.05	7/224 (3.1)	NA^*^
ANB (°)	−2.02	+0.09 (+0.03, +0.15)	1.04	0.09	8/224 (3.6)	95.5
*Y*-axis angle (°)	+1.53	−0.02 (−0.11, +0.07)	1.53	0.01	5/224 (2.2)	98.7
U1-NA (°)	−6.08	+0.37 (+0.22, +0.52)	1.46	0.25	13/224 (5.8)	93.9
L1-NB (°)	−4.45	+0.24 (+0.10, +0.38)	1.57	0.15	11/224 (4.9)	94.6
U1-L1 (°)	+9.20	−0.69 (−0.91, −0.47)	2.14	0.32	15/224 (6.7)	92.5
U1-NA (mm)	−3.21	+0.10 (+0.05, +0.15)	0.50	0.20	12/224 (5.4)	96.9
L1-NB (mm)	−2.64	+0.08 (+0.03, +0.13)	0.56	0.14	10/224 (4.5)	97.0
U1-L1 (mm)	+0.88	−0.06 (−0.11, −0.01)	0.67	0.09	9/224 (4.0)	93.2
Nasolabial angle (°)	+9.42	−0.55 (−0.82, −0.28)	2.49	0.22	14/224 (6.3)	94.2
Mentolabial angle (°)	+7.63	−0.40 (−0.69, −0.11)	2.68	0.15	12/224 (5.4)	94.8
UL–E line (mm)	−2.04	+0.08 (+0.05, +0.11)	0.39	0.21	15/224 (6.7)	96.1
LL–E line (mm)	−1.12	+0.06 (+0.03, +0.09)	0.44	0.14	13/224 (5.8)	94.6
Soft-tissue chin thickness (mm)	+2.41	−0.09 (−0.14, −0.04)	0.61	0.15	11/224 (4.9)	96.3

Every value in this table was measured on a lateral cephalogram: T0 and T1 on the orthogonally synthesized cephalograms reformatted from the corresponding CBCT volumes, T2 on the conventional cephalogram taken at the retention review. The T0–T1 changes therefore differ slightly from the three-dimensional values in Table 2. Combined SDD = 1.96 × √(DE^2^T1 + DE^2^T2), the smallest change in an individual that can be separated from method error with 95% confidence (Supplementary Table S2). Maintained change = |T2 – T0|/|T1 – T0| × 100. The qualitative stability categories reported previously have been withdrawn and replaced by this measurement-error criterion.

^*^SNA and SNB did not change significantly between T0 and T1, so the proportion of a treatment change that is maintained is undefined for them; their T1–T2 values are listed to show that the basal relationship also remained unchanged. No variable reached its combined SDD, and for every variable the confidence limit furthest from zero also lay below the SDD. A group mean below the SDD indicates that the average change cannot be separated from method error; it is not a formal demonstration of equivalence, and it does not describe the individual patient, which is why the individual-level exceedance is given in the penultimate column. The percentages in the final column are retained for continuity with the earlier literature and should be read beside the SDD column: a mean displacement of 0.06 mm, measured by a method whose 95% error band is 0.44 mm, supports a statement about the absence of detectable relapse rather than a claim that exactly 94.6% of the change persisted.

The group mean, however, is not the patient. Between 2.2% (*Y*-axis angle, 5 of 224) and 6.7% (interincisal angle and UL–E, 15 of 224) of individuals showed a T1–T2 change that exceeded the combined SDD in the relapse direction (Table 3, Figure 4B). The variables with the highest individual exceedance were those most directly manipulated by treatment: incisor inclination, the interincisal angle and upper-lip position. The correct reading is therefore that the immediate dentoalveolar and soft-tissue result was retained through the first year of retention at group level, with no mean change distinguishable from the error of the method, while a small minority of patients underwent measurable rebound. Longer follow-up is required to establish late stability.

### Hard–soft tissue associations and their persistence

**Figure 5:**
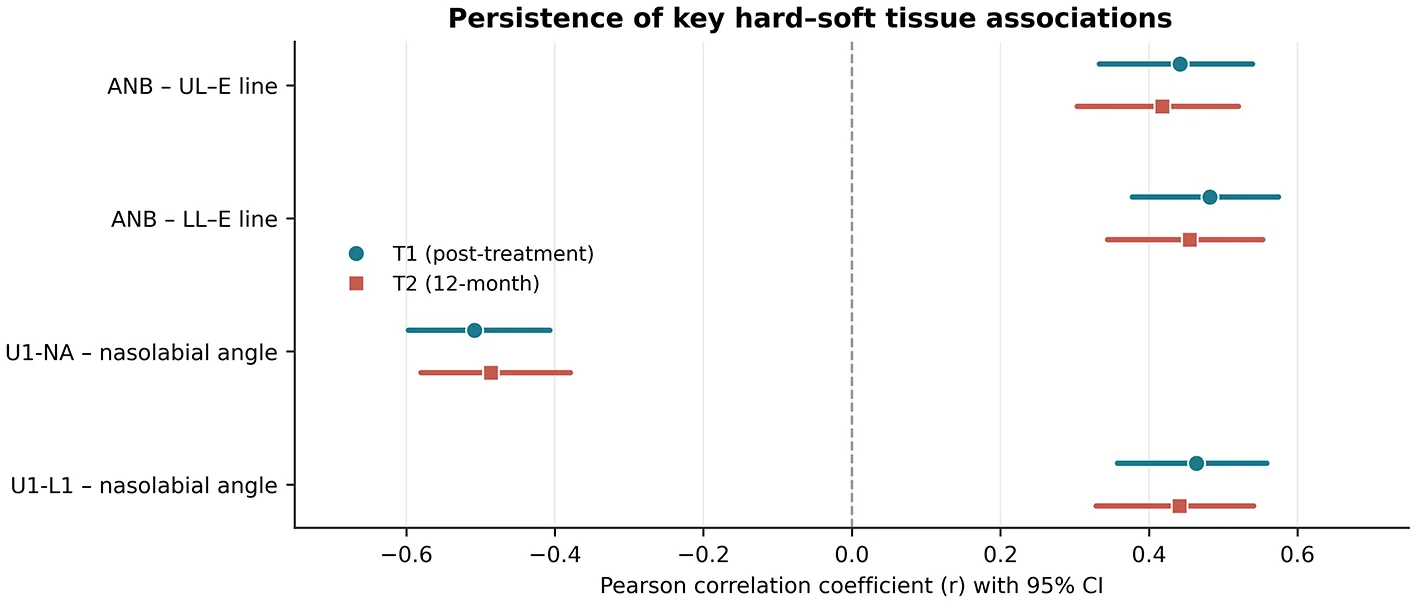
The four hard–soft tissue correlations that were significant after Bonferroni correction, shown as Pearson coefficients with 95% CIs at T1 (post-treatment) and at the 12-month retention review (T2), demonstrating persistence of the associations. Because Pearson coefficients are dimensionless and the same-scan agreement between the volumetric and the cephalometric measurement was high (Supplementary Table S3), the two sets of coefficients are compared descriptively and were not tested against one another.

Four of the 25 cross-sectional correlations were significant at T1 and remained directionally consistent at 12 months (Figure 5; full matrix in Supplementary Table S6). Pearson coefficients are dimensionless, and the same-scan agreement documented in Supplementary Table S3 was high, so the T1 and T2 coefficients are compared descriptively rather than tested against one another. ANB correlated positively with UL–E (T1 *r* = 0.442; T2 *r* = 0.418) and LL–E (T1 *r* = 0.482; T2 *r* = 0.455), indicating that a larger residual sagittal discrepancy was associated with greater lip protrusion relative to the E-plane. U1-NA correlated negatively (T1 *r* = −0.508; T2 *r* = −0.486) and U1-L1 positively (T1 *r* = 0.464; T2 *r* = 0.441) with the nasolabial angle, showing a persistent relationship between maxillary incisor position and the nasolabial profile. Spearman analyses gave concordant results.

### Coupling of hard- and soft-tissue change

**Figure 6:**
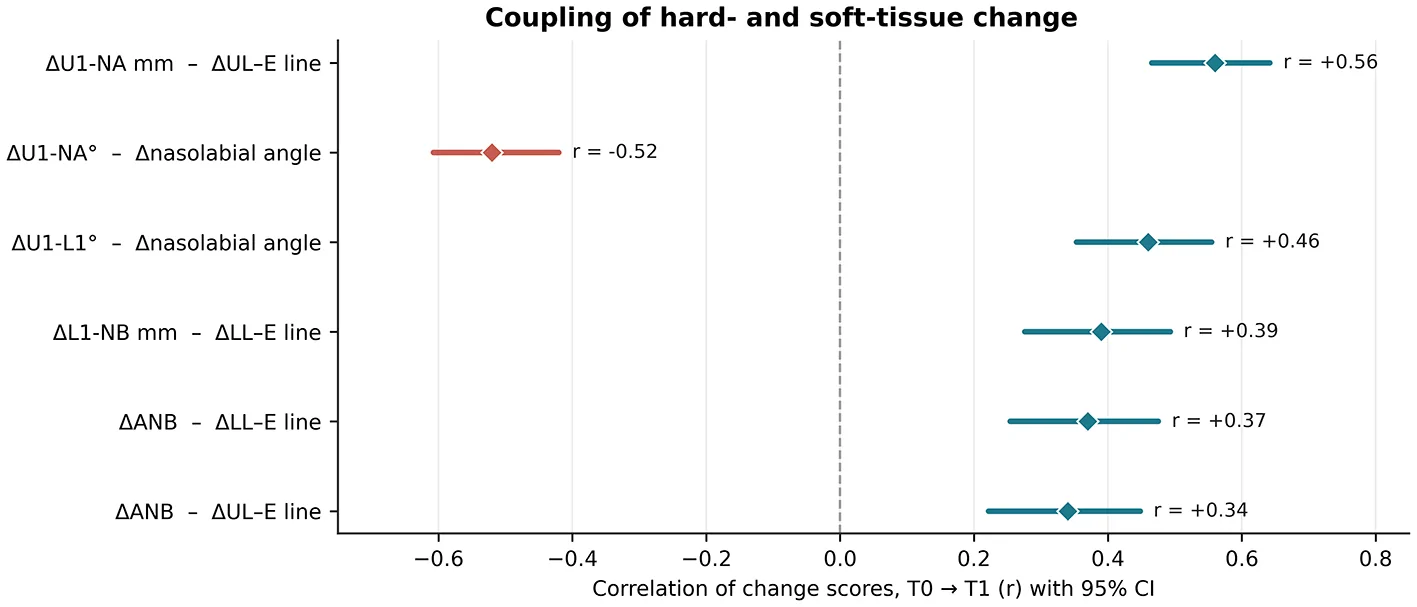
Correlations between hard- and soft-tissue change scores (Δ T0–T1), with 95% *CIs*, showing that the magnitude of dentoalveolar change was coupled to the magnitude of soft-tissue change.

Change-score analyses showed that the magnitude of hard-tissue change was associated with the magnitude of soft-tissue change (Figure 6 and Supplementary Table S7). The corresponding full post-treatment scatter plots for the cross-sectional analyses are provided in Supplementary Figures S1–S5. Greater maxillary incisor retraction accompanied greater upper-lip retraction (ΔU1-NA mm vs. ΔUL–E, *r* = 0.56) and a larger nasolabial-angle increase (ΔU1-NA° vs. Δ nasolabial angle, *r* = −0.52), while greater mandibular incisor retraction accompanied greater lower-lip retraction (ΔL1-NB mm vs. ΔLL–E, *r* = 0.39; all *P* < 0.001). In multivariable models (Table 4; full output in Supplementary Table S8), maxillary incisor retraction remained an independent correlate of nasolabial-angle increase (*B* = −0.96° per degree of ΔU1-NA; 95% *CI* −1.20 to −0.72; standardized β = −0.36) and of upper-lip retraction (*B* = +0.24 mm per millimeter of ΔU1-NA; 95% *CI* +0.18 to +0.30; β = +0.44) after adjustment for age, sex, the baseline value of the outcome, extraction status and treatment duration. Mandibular incisor retraction remained independently associated with lower-lip retraction (*B* = +0.16; 95% *CI* +0.10 to +0.22; β = +0.31), and a residual sagittal discrepancy contributed to it as well (ΔANB: *B* = +0.11; 95% *CI* +0.04 to +0.18). Extraction treatment and a more protrusive baseline were each associated with greater lip retraction. The coefficients previously reported as β in this cohort were unstandardized, and both quantities are now given separately.

**Table 4:** Multivariable linear regression for soft-tissue change (T0–T1), *n* = 236.

Outcome (Δ T0–T1)	Key predictor	*B* (95% *CI*)	β	*P*	VIF
Δ Nasolabial angle (°)	ΔU1-NA angle (°)	−0.96 (−1.20, −0.72)	−0.36	< 0.001	1.44
	Extraction treatment	+2.08 (+0.54, +3.62)	+0.12	0.008	1.71
	Baseline nasolabial angle	−0.29 (−0.40, −0.18)	−0.33	< 0.001	1.18
	Model summary	*R*^2^ = 0.356; adjusted *R*^2^ = 0.339	*F*_(6, 229)_ = 21.10	< 0.001
Δ UL–E line (mm)	ΔU1-NA (mm)	+0.24 (+0.18, +0.30)	+0.44	< 0.001	1.57
	Extraction treatment	−0.19 (−0.30, −0.08)	−0.20	0.001	1.76
	Baseline UL–E line	−0.36 (−0.48, −0.24)	−0.25	< 0.001	1.14
	Model summary	*R*^2^ = 0.412; adjusted *R*^2^ = 0.397	*F*_(6, 229)_ = 26.74	< 0.001
Δ LL–E line (mm)	ΔL1-NB (mm)	+0.16 (+0.10, +0.22)	+0.31	< 0.001	1.48
	ΔANB (°)	+0.11 (+0.04, +0.18)	+0.17	0.002	1.36
	Baseline LL–E line	−0.29 (−0.42, −0.16)	−0.22	< 0.001	1.17
	Model summary	*R*^2^ = 0.278; adjusted *R*^2^ = 0.256	*F*_(7, 228)_ = 12.54	< 0.001
Δ Mentolabial angle (°)	ΔL1-NB angle (°)	−0.54 (−0.86, −0.22)	−0.19	0.001	1.42
	Baseline mentolabial angle	−0.27 (−0.38, −0.16)	−0.31	< 0.001	1.15
	Model summary	*R*^2^ = 0.168; adjusted *R*^2^ = 0.146	*F*_(6, 229)_ = 7.71	< 0.001
	Δ Chin thickness (mm)	Extraction treatment	+0.17 (−0.02, +0.36)	+0.10	0.078	1.69
Baseline chin thickness		−0.31 (−0.39, −0.23)	−0.46	< 0.001	1.20
Model summary		*R*^2^ = 0.231; adjusted *R*^2^ = 0.211	*F*_(6, 229)_ = 11.46	< 0.001

Every model was adjusted for age, sex, the baseline value of the outcome, extraction status and treatment duration; the key predictors are shown here and the complete models, with all covariates and with the residual and influence diagnostics, are given in Supplementary Table S8. Anchorage is not entered here because every TAD and every elastics patient was an extraction patient, so that extraction status is the exact sum of the two anchorage indicators; anchorage is examined separately, within the 168 extraction patients, in Supplementary Table S11. *B* is the unstandardized coefficient and β the standardized coefficient; VIF is the variance inflation factor.

The 95% confidence interval for ΔANB in the ΔLL–E model excludes zero, indicating that a residual sagittal discrepancy contributed to lower-lip position independently of mandibular incisor retraction. The maximum VIF across all five models was 1.76 and the maximum tolerance-based collinearity was correspondingly low, so no predictor was materially collinear. Residual and influence diagnostics (Durbin–Watson 1.91–2.07; Shapiro–Wilk *P* ≥ 0.073; Breusch–Pagan *P* ≥ 0.241; maximum Cook's distance 0.015; maximum leverage 0.059) are reported for every model in Supplementary Table S8; residual-vs.-fitted and normal probability plots were inspected for each model and showed no departure from the assumptions. Because treatment was allocated by clinical indication, the extraction coefficient combines the effect of the mechanics with the effect of the indication for them and is not a causal estimate.

Model fit was modest rather than strong. Adjusted *R*^2^ ranged from 0.146 for Δ mentolabial angle to 0.397 for ΔUL–E, all five models being significant overall (*F* statistics and degrees of freedom in Table 4), so that the upper lip was the best explained outcome and the labiomental fold the least. The maximum variance inflation factor across all models was 1.76, giving no evidence of problematic multicollinearity, and the residual diagnostics were unremarkable (Durbin–Watson 1.91 to 2.07, Shapiro–Wilk *P* ≥ 0.073, Breusch–Pagan *P* ≥ 0.241, maximum Cook's distance 0.015; Supplementary Table S8). It is worth setting the adjusted slope beside the crude one: the mean upper lip moved 2.08 mm for a mean incisor retraction of 3.25 mm, a ratio of 0.64, whereas the within-cohort slope after adjustment for baseline lip position and the remaining covariates is 0.24 mm per millimeter. A ratio of mean changes and a regression slope answer different questions, and neither licenses a prediction for an individual face.

### Subgroup and sensitivity analyses

The extraction group showed larger immediate dentoalveolar and soft-tissue change than the non-extraction group (for example, ΔUL–E −2.28 vs. −1.58 mm; Δ nasolabial angle +11.1° vs. +5.9°; both *P* < 0.001), while 12-month stability did not differ materially between them (Supplementary Table S9). These are group-associated differences: extraction was chosen for the patients with the larger discrepancy, and the two groups differed at baseline by construction. Within the 168 extraction patients, maxillary incisor retraction was greater in those treated with TADs than in those treated with Class II elastics (ΔU1-NA mm −3.9 vs. −3.4 mm; *P* < 0.001), whereas mandibular incisor change was somewhat greater with elastics (Supplementary Table S10). In regression models restricted to these patients, anchorage category was associated with ΔUL–E (TAD vs. elastics *B* = −0.10 mm; 95% *CI* −0.19 to −0.01; *P* = 0.031) and with ΔLL–E (*B* = +0.12 mm; 95% *CI* +0.03 to +0.21; *P* = 0.010), but not with the angular soft-tissue outcomes (*P* ≥ 0.235; Supplementary Table S11). The three retention regimens showed no detectable difference in any T1–T2 outcome (all omnibus *P* ≥ 0.841), and adding retainer type to the principal models changed the main hard-tissue coefficients by no more than 5.9% (Supplementary Table S12). Because anchorage and retainer type were assigned by indication, none of these comparisons is interpreted as a treatment effect.

## Discussion

In this large cohort of non-growing adults with Angle Class II Division 1 malocclusion, comprehensive treatment was accompanied by predominantly dentoalveolar correction and by coupled soft-tissue change that was retained at 1 year at group level, no mean change reaching the error of the method by which it was measured. The stability of SNA and SNB indicates that the basal sagittal relationship was essentially unchanged, as expected in skeletally mature patients; correction therefore manifested mainly through retraction and uprighting of the previously proclined incisors. This distinction is important clinically because it helps separate realistic adult camouflage effects from skeletal effects that would require growth modification or orthognathic surgery. The concurrent reduction in lip protrusion and increase in nasolabial and mentolabial angles suggest that the dentoalveolar correction translated into visible profile changes, particularly in the lip–nose and lip–chin regions (5, 7, 17, 18).

The principal advance over earlier reports is the move from association between final morphologies to coupling between changes. The change-score and adjusted analyses showed that the amount of maxillary incisor retraction was independently related to the amount of nasolabial-angle increase and upper-lip retraction, and mandibular incisor retraction to lower-lip retraction, after accounting for age, sex, baseline morphology and treatment mechanics. This directly addresses the common criticism that post-treatment correlations show only that final shape is associated with final shape; here, patients who underwent greater dentoalveolar change also underwent greater soft-tissue change. The findings are therefore biologically coherent and useful for counseling, while remaining non-causal, since there was no untreated or alternative-treatment control group.

Coupling, however, is not prediction, and the distinction deserves more weight than it usually receives. The change-score correlations reported here lie between *r* = 0.39 and *r* = 0.56, so that between roughly 15% and 31% of the variance in soft-tissue change is shared with the dentoalveolar change and the greater part of it is not. The adjusted models, which additionally carry age, sex, baseline morphology, extraction status and treatment duration, do not close that gap: adjusted *R*^2^ reaches 0.397 for the upper lip and falls to 0.146 for the labiomental fold. A clinician may therefore tell an adult patient that, on average, retracting the maxillary incisors will open the nasolabial angle and carry the upper lip toward the aesthetic plane, and may quote the mean figures reported here. That same clinician cannot say how much of the change will occur in this patient's face. Lip thickness, lip strain, lip competence at rest, perioral muscle tone and the vertical relation of the incisal edge to stomion all intervene between the tooth and the vermilion, and none of them was measured (10, 12). The gap between the crude ratio of mean changes, 0.64 mm of upper-lip movement per millimeter of incisor retraction, and the adjusted within-cohort slope of 0.24 mm per millimeter makes the same point arithmetically: the proportionality that seems evident in the group means is not the quantity that governs the individual response.

The soft-tissue pattern, an increased nasolabial angle, lips retracted toward the aesthetic plane, and an increased mentolabial sulcus angle, mirrors repeated observations after retraction of protrusive incisors, particularly with premolar extraction (7, 8, 29–32). We deliberately describe these as profile changes consistent with reduced lip protrusion rather than as aesthetic “improvement”, because patient-reported or expert aesthetic outcomes were not assessed. The modest increase in the *Y*-axis angle also deserves attention. It suggests a small clockwise tendency of the facial axis; therefore, the more favorable chin contour is better explained by reduced lip prominence and an altered labiomental relationship than by anterior mandibular rotation. The vertical facial pattern is known to modify how a given amount of incisor retraction is expressed in the profile, being least favorable in long-face patients, and it was not recorded here (33); the conservative reading is therefore the defensible one.

The literature on this question is not unanimous, and it would misrepresent it to present our findings as though it were. One tradition, beginning with the lip-thickness and lip-strain analyses of the 1980s, reports close and fairly proportional coupling between maxillary incisor retraction and upper-lip retraction, with ratios approaching 1:0.7 in thin, strained lips (9, 10). A second tradition denies that any usable proportion exists. Hodges and colleagues found that pre-treatment cephalometric variables predicted only a small fraction of the lip response to four premolar extractions and concluded that individual prediction was not clinically useful (34). Kim and co-workers, studying precisely the population examined here, adults with Class II Division 1 malocclusion treated by maximum anterior retraction on miniscrews, titled their paper on the unpredictability of the soft-tissue response (11). Kuhn and colleagues (12) referenced tooth movement to a rough-surfaced palatal implant, so that the hard-tissue displacement was known with negligible error, and still found that the type of incisor movement, bodily retraction as against tipping, altered the lip response for the same displacement of the incisal edge. That last observation is decisive, because it excludes measurement error of the predictor as the explanation for the unexplained variance. Our results sit between these positions rather than beside either. The coupling we observe is real, reproducible across two independent hard-tissue predictors and robust to adjustment; the residual variance we observe is large, and is consistent with the pessimistic tradition. Both statements are true at once, and the apparent conflict in the earlier literature is at least partly a conflict between describing a group and predicting an individual.

A biological reading is available and worth stating, because it explains why the coefficients differ across the soft-tissue variables. The upper lip is not attached to the maxillary incisor; it rests against it. Where the lip is thin and stretched over a prominent incisor, the incisor carries the lip and retraction unloads it almost point for point, which is the circumstance in which high ratios are reported (10, 34). Where the lip is thick, or where the lips are already competent at rest, much of the retraction is absorbed by a change in vermilion shape and by redistribution of soft-tissue bulk rather than by displacement of the lip as a whole, and the ratio falls. The nasolabial angle behaves differently again, because its upper limb, the columella, is fixed to the nose and does not move with the dentition, so that the angle opens whenever the lower limb rotates; this predicts, and our data show, that the change in U1-NA angle is the better correlate of nasolabial change. The mentolabial angle and the apparent increase in soft-tissue chin thickness are best read as consequences of lower-lip repositioning and of relief of mentalis strain rather than as any change in the chin, since hard-tissue pogonion cannot move in a non-growing adult treated without surgery. The point is worth making explicitly, because an increase in tabulated chin thickness can otherwise be misread as tissue growth.

Clinically, the 12-month data are encouraging but must be read at two levels. Between 92.5% and 98.7% of the T0–T1 change was still present at 1 year, and no variable moved by as much as its combined smallest detectable difference, so the group-mean rebound lies within the noise of the method rather than above it (25, 26). At the level of the individual, however, between 2.2% and 6.7% of patients crossed that threshold in the relapse direction. Group-level stability is not stability in every patient, and the variables in which individuals most often crossed the threshold, incisor inclination, the interincisal angle and upper-lip position, are exactly those that treatment moved furthest. Stability did not differ materially between extraction and non-extraction patients. The subgroup analyses add nuance for treatment planning, the extraction and TAD groups showing larger immediate dentoalveolar and lip changes, while short-term retention stability was broadly similar across mechanics. This pattern is plausible because TADs improve maxillary anchorage control, whereas Class II elastics distribute their reciprocal effects more strongly to the mandibular dentition, a contrast that the anchorage sensitivity models reproduce and that network meta-analysis of anchorage systems also supports (35). The dentoalveolar changes reported here are of the order achievable by camouflage, and patients whose discrepancy lies beyond that range are those for whom surgical correction continues to be discussed (36, 37). Together, these results give adult patients more realistic expectations: fixed-appliance treatment can produce meaningful profile change, but the magnitude depends on the amount and direction of incisor movement and on the treatment mechanics used.

The stability findings rest on a different evidential footing from the treatment-change findings, and deserve a paragraph of their own. The T0 to T1 comparison uses two three-dimensional volumes acquired on one machine under one protocol. The T1 to T2 comparison uses two lateral cephalograms, one of them synthesized from a volume and one acquired conventionally. Reformatting the T1 volume removes the grossest source of incomparability, the difference between a three-dimensional and a projected measurement, and the same-scan substudy shows what that step costs: a bias no larger than 0.18° or 0.06 mm, with limits of agreement inside the combined SDD for every variable. What reformatting cannot remove is the residual difference between a synthesized and a conventional film in contrast and resolution, the reproducibility of head position in a cephalostat, and the fact that relaxed lip posture is a physiological and not a geometric state. We have not assumed that these residual errors are purely random. A systematic component cannot be excluded, and a systematic component could in principle push the T1–T2 difference in either direction; this is precisely why the same-scan bias was measured rather than argued about, and why the combined SDD, rather than the percentage maintained, carries the conclusion. It remains an absence of detectable relapse and not a demonstration of true stability. A percentage of 94.6% sounds precise; what it rests on is a mean displacement of 0.06 mm measured by a method whose 95% error band is 0.44 mm. Whether these patients are still stable at 5 or 10 years cannot be answered here, and the retention literature indicates that the question is a real one (38).

Generalizability is limited in four ways that compound rather than add. The cohort is single-center, so it reflects one department's diagnostic thresholds, mechanics and retention rule. It is exclusively Chinese, and ethnic variation in lip thickness, nasal projection and soft-tissue drape is large enough to alter the coupling ratios reported here. It is young, with a mean age of 21.4 years, so the soft tissues retain a tone they will not retain at forty, and the findings should not be transferred to middle-aged adults without comment. Finally, the dispersion of the paired differences for several variables in this cohort is very low, and because Cohen's *dz* is the mean change divided by the standard deviation of those differences, it is that low dispersion, and not an unusually large treatment effect, that produces standardized effect sizes such as *dz* = 7.86 for UL–E. The same narrow range attenuates the intraclass correlation for UL–E in the cross-modality substudy, where the Bland and Altman limits are the more informative statistic. Those effect sizes are arithmetically correct and are reported unchanged, but the mean changes with their confidence intervals are the quantities that transfer.

Treatment in this cohort was allocated by clinical indication and not at random, and the consequence is specific. The patients sent for extraction, and within them the patients who received skeletal anchorage, were the patients with the larger overjet, the greater crowding and the more convex profile; they then underwent the larger dentoalveolar and soft-tissue change. Part of the extraction coefficient in Table 4 is an effect of the mechanics and part is an effect of the indication for those mechanics, and no adjustment performed on observational data can locate the boundary between them. We adjusted for the covariates recorded and stratified by extraction status and by anchorage category, and we have deliberately not attempted to quantify the residual confounding, since converting continuous regression coefficients to a risk-ratio scale for that purpose would introduce assumptions we cannot verify. Residual and unmeasured confounding therefore cannot be excluded. Several variables known to matter were not recorded: the vertical skeletal pattern and facial type, the baseline severity of the skeletal discrepancy beyond ANB, lip thickness and lip competence at rest, and body mass index and its change during treatment. Each could plausibly influence both the decision to extract and the soft-tissue response, which is the definition of a confounder. The one vertical proxy we did record, the *Y*-axis angle, showed no significant correlation with any soft-tissue variable at either time point (Supplementary Table S6), which bounds but does not eliminate the concern within the restricted range of severity that the inclusion criteria admitted. A prospective study that records these variables, and that compares concurrent treatment alternatives rather than treated patients against nothing, is the design that would settle the question.

Strengths include a comparatively large, consecutively recruited and well-defined adult cohort; clinically archived CBCT assessment at the two time points at which three-dimensional information is actually used; modality-matched measurement at follow-up with reliability established separately for each image type; explicit reporting of method error, of the smallest detectable difference and of individual-level exceedance; change-score, adjusted and subgroup analyses; and full reporting of model fit, collinearity and residual diagnostics. Several limitations remain and constrain interpretation. The single-center observational pre–post design without an untreated or alternative-treatment control group cannot establish causality or exclude factors unrelated to treatment, although growth is improbable in adults. The cohort combined extraction, non-extraction, TAD and elastic mechanics; although we adjusted for extraction status and analyzed anchorage only within extraction patients, residual confounding by indication remains. Soft-tissue measurement is sensitive to facial posture, lip position and head support even when acquisition is standardized, and relaxed lip posture can be instructed but not guaranteed. No research-only imaging was requested at any point, so the 12-month analysis rests on a two-dimensional record acquired for clinical purposes rather than on a uniform prospective T2 CBCT protocol; projecting the T1 volume into the same geometry and measuring the cost of that projection reduces, but does not abolish, the resulting measurement heterogeneity, and the stability conclusions are correspondingly weaker than the treatment-change conclusions. The measurement-error criterion used to judge stability was introduced after the primary analysis rather than pre-specified, and a group mean lying below the smallest detectable difference is an absence of detectable change rather than a formal demonstration of equivalence; no equivalence margin could be justified from published clinical standards, so none was imposed. Follow-up extended to 12 months only, so longer-term stability beyond the first year remains unknown. Retention regimen was likewise assigned by indication, so the similarity of stability across retainer types cannot be read as evidence that retainer type does not matter; the sensitivity analysis in Supplementary Table S12 is a check on the coefficients of interest and not an estimate of a retention effect. Finally, the cohort comprised young Chinese adults treated at one center, so generalization to other populations, older or growing patients, other malocclusions or other appliances requires further study. The findings should be read as describing association, change and short-term stability rather than causal benefit, and warrant confirmation in prospective controlled studies.

## Conclusion

In non-growing adults with Angle Class II Division 1 malocclusion, CBCT-based pre/post assessment showed that comprehensive orthodontic treatment was accompanied by dentoalveolar correction and coupled soft-tissue profile change. Modality-matched follow-up at 12 months showed no mean change that reached the smallest detectable difference of any variable, so the immediate result was maintained through the first year of retention as far as the method can resolve, although a small minority of individuals crossed that threshold. Maxillary incisor retraction was independently associated with nasolabial-angle increase and upper-lip retraction, but the coupling explained a minority of the between-patient variance, so individual soft-tissue outcomes remain difficult to predict. Given the observational, uncontrolled design and the two-dimensional follow-up record, these results describe change, association and short-term stability rather than causal improvement, and should be confirmed in prospective controlled studies with longer follow-up.

## Data Availability

The original contributions presented in the study are included in the article/Supplementary material, further inquiries can be directed to the corresponding author.
