# Supplementary material for: Hard- and soft-tissue changes after adult Class II Division 1 orthodontic treatment: a retrospective CBCT-based observational study
Source: Front Med (Lausanne). 2026 Aug 6;13:1911048. doi: 10.3389/fmed.2026.1911048 (PMC13489871; doi:10.3389/fmed.2026.1911048)
Supplement: Supplementary file 1 [file Supplementary_File_1.docx]

**Supplementary Material**

**Hard- and Soft-Tissue Changes After Adult Class II Division 1 Orthodontic Treatment: A Retrospective CBCT-Based Observational Study**

**Supplementary Table S1. Landmark and measurement definitions**

| **Variable** | **Definition** | **Unit** |
| --- | --- | --- |
| SNA | Angle formed by the sella–nasion and nasion–A point lines | ° |
| SNB | Angle formed by the sella–nasion and nasion–B point lines | ° |
| ANB | Difference between SNA and SNB | ° |
| Y-axis angle | Angle between the sella–gnathion line and the Frankfort horizontal plane | ° |
| U1-NA (°) | Angle between the maxillary central incisor long axis and the NA line | ° |
| U1-NA (mm) | Perpendicular distance from the maxillary incisor edge to the NA line | mm |
| L1-NB (°) | Angle between the mandibular central incisor long axis and the NB line | ° |
| L1-NB (mm) | Perpendicular distance from the mandibular incisor edge to the NB line | mm |
| U1-L1 (°) | Interincisal angle between the maxillary and mandibular incisor long axes | ° |
| U1-L1 (mm) | Shortest distance between the maxillary and mandibular central incisor edges on the mid-sagittal reconstruction | mm |
| Nasolabial angle | Angle formed by the columella, subnasale and the upper-lip tangent | ° |
| Mentolabial angle | Angle of the lower lip, mentolabial sulcus and soft-tissue chin contour | ° |
| UL–E line | Distance from the upper lip to the Ricketts aesthetic (E) plane | mm |
| LL–E line | Distance from the lower lip to the Ricketts aesthetic (E) plane | mm |
| Soft-tissue chin thickness | Distance between hard-tissue pogonion and soft-tissue pogonion | mm |

*The same definitions were applied without modification to the three-dimensional reconstructions, to the orthogonally synthesised lateral cephalograms reformatted from the T0 and T1 volumes, and to the conventional lateral cephalograms acquired at the 12-month retention review.*

**Supplementary Table S2. Measurement reliability, method error and the combined smallest detectable difference**

*Panel A. Three-dimensional CBCT measurements (T0 and T1), used for the treatment-change analysis (30 scans re-measured).*

| **Variable** | **Intra-examiner ICC** | **Inter-examiner ICC** | **Dahlberg error** | **Systematic error, P** |
| --- | --- | --- | --- | --- |
| SNA (°) | 0.982 | 0.965 | 0.42° | 0.684 |
| SNB (°) | 0.980 | 0.962 | 0.45° | 0.591 |
| ANB (°) | 0.976 | 0.958 | 0.36° | 0.744 |
| Y-axis angle (°) | 0.971 | 0.949 | 0.51° | 0.433 |
| U1-NA (°) | 0.984 | 0.966 | 0.48° | 0.528 |
| L1-NB (°) | 0.979 | 0.954 | 0.52° | 0.617 |
| U1-L1 (°) | 0.975 | 0.951 | 0.73° | 0.482 |
| U1-NA (mm) | 0.988 | 0.972 | 0.16 mm | 0.635 |
| L1-NB (mm) | 0.985 | 0.968 | 0.18 mm | 0.573 |
| U1-L1 (mm) | 0.964 | 0.941 | 0.22 mm | 0.491 |
| Nasolabial angle (°) | 0.961 | 0.936 | 0.82° | 0.664 |
| Mentolabial angle (°) | 0.958 | 0.931 | 0.89° | 0.702 |
| UL–E line (mm) | 0.982 | 0.963 | 0.12 mm | 0.811 |
| LL–E line (mm) | 0.979 | 0.959 | 0.14 mm | 0.768 |
| Soft-tissue chin thickness (mm) | 0.973 | 0.948 | 0.20 mm | 0.588 |

*Panel B. Cephalometric measurements used for the stability analysis: the orthogonally synthesised T1 cephalogram and the conventional T2 cephalogram (30 records of each type re-measured).*

| **Variable** | **Synthesised T1: Dahlberg error** | **T2 intra-examiner ICC** | **T2 inter-examiner ICC** | **T2 Dahlberg error** | **Combined SDD** |
| --- | --- | --- | --- | --- | --- |
| SNA (°) | 0.44 | 0.978 | 0.961 | 0.48 | 1.28° |
| SNB (°) | 0.46 | 0.976 | 0.958 | 0.50 | 1.33° |
| ANB (°) | 0.35 | 0.972 | 0.951 | 0.40 | 1.04° |
| Y-axis angle (°) | 0.52 | 0.968 | 0.944 | 0.58 | 1.53° |
| U1-NA (°) | 0.50 | 0.979 | 0.957 | 0.55 | 1.46° |
| L1-NB (°) | 0.54 | 0.974 | 0.950 | 0.59 | 1.57° |
| U1-L1 (°) | 0.74 | 0.966 | 0.939 | 0.80 | 2.14° |
| U1-NA (mm) | 0.17 | 0.982 | 0.964 | 0.19 | 0.50 mm |
| L1-NB (mm) | 0.19 | 0.980 | 0.960 | 0.21 | 0.56 mm |
| U1-L1 (mm) | 0.23 | 0.958 | 0.928 | 0.25 | 0.67 mm |
| Nasolabial angle (°) | 0.84 | 0.954 | 0.925 | 0.95 | 2.49° |
| Mentolabial angle (°) | 0.91 | 0.951 | 0.921 | 1.02 | 2.68° |
| UL–E line (mm) | 0.13 | 0.977 | 0.953 | 0.15 | 0.39 mm |
| LL–E line (mm) | 0.15 | 0.974 | 0.949 | 0.17 | 0.44 mm |
| Soft-tissue chin thickness (mm) | 0.21 | 0.969 | 0.940 | 0.23 | 0.61 mm |

*ICC, intraclass correlation coefficient (two-way mixed effects, absolute agreement). Dahlberg errors are expressed in the unit of the variable concerned. On the conventional T2 cephalograms, intra-examiner ICCs ranged from 0.951 to 0.982 and inter-examiner ICCs from 0.921 to 0.964; Dahlberg errors were no greater than 1.02° for angular and 0.25 mm for linear variables, with no systematic difference between repeated measurements (all P > 0.05). Combined SDD = 1.96 × √(DE²T1 + DE²T2), where DE is the Dahlberg error of the record concerned; this is the smallest change in an individual that can be separated from method error with 95% confidence and is the criterion used in Table 3 and Figure 6. Reliability was assessed separately for each image type because reliability established on a three-dimensional volume does not transfer to a projection.*

**Supplementary Table S3. Same-scan agreement between three-dimensional and CBCT-derived cephalometric measurements (n = 40)**

| **Variable** | **3D, mean ± SD** | **Synthesised 2D, mean ± SD** | **Mean bias** | **95% limits of agreement** | **ICC** |
| --- | --- | --- | --- | --- | --- |
| SNA (°) | 80.34 ± 8.11 | 80.27 ± 8.06 | +0.07 | −0.70 to +0.84 | 0.996 |
| SNB (°) | 76.22 ± 7.66 | 76.16 ± 7.59 | +0.06 | −0.74 to +0.86 | 0.995 |
| ANB (°) | 5.42 ± 0.57 | 5.40 ± 0.59 | +0.02 | −0.48 to +0.52 | 0.990 |
| Y-axis angle (°) | 68.11 ± 6.90 | 68.18 ± 6.84 | −0.07 | −0.92 to +0.78 | 0.994 |
| U1-NA (°) | 26.14 ± 2.71 | 26.04 ± 2.75 | +0.10 | −1.02 to +1.22 | 0.986 |
| L1-NB (°) | 27.31 ± 2.78 | 27.39 ± 2.82 | −0.08 | −1.20 to +1.04 | 0.984 |
| U1-L1 (°) | 126.48 ± 12.51 | 126.36 ± 12.56 | +0.12 | −1.40 to +1.64 | 0.992 |
| U1-NA (mm) | 4.07 ± 0.43 | 4.03 ± 0.45 | +0.04 | −0.28 to +0.36 | 0.971 |
| L1-NB (mm) | 5.42 ± 0.56 | 5.38 ± 0.58 | +0.04 | −0.33 to +0.41 | 0.968 |
| U1-L1 (mm) | 13.49 ± 1.33 | 13.45 ± 1.35 | +0.04 | −0.41 to +0.49 | 0.963 |
| Nasolabial angle (°) | 98.41 ± 9.92 | 98.23 ± 9.86 | +0.18 | −1.76 to +2.12 | 0.982 |
| Mentolabial angle (°) | 83.17 ± 8.41 | 83.01 ± 8.35 | +0.16 | −1.94 to +2.26 | 0.978 |
| UL–E line (mm) | 0.53 ± 0.06 | 0.56 ± 0.08 | −0.03 | −0.24 to +0.18 | 0.905 |
| LL–E line (mm) | 2.43 ± 0.27 | 2.47 ± 0.29 | −0.04 | −0.31 to +0.23 | 0.948 |
| Soft-tissue chin thickness (mm) | 13.51 ± 1.39 | 13.45 ± 1.36 | +0.06 | −0.36 to +0.48 | 0.981 |

*In forty randomly selected patients the same T1 scan was measured twice, once volumetrically and once on the orthogonally synthesised lateral cephalogram reformatted from it, so that any difference is attributable to the projection alone and cannot reflect repositioning, biological change or the passage of time. Bias is defined as the three-dimensional value minus the cephalometric value. No mean bias exceeded 0.18° or 0.07 mm, and for every variable the limits of agreement lay within the combined SDD of Supplementary Table S2. Regression of the difference on the average gave no evidence of proportional bias for any variable (all P > 0.05), and Bland and Altman plots were inspected for each. The intraclass correlation for UL–E is the lowest in the table (0.905) because that variable has a very narrow between-patient range in this cohort, which attenuates the coefficient; for such variables the bias and the limits of agreement are the informative statistics, and both are small. None of the observed biases is clinically meaningful, each being well below the smallest detectable difference of the corresponding variable.*

**Supplementary Table S4. Treatment mechanics, retention regimen and adherence**

*Panel A. Mechanics.*

| **Characteristic** | **n (%)** |
| --- | --- |
| Non-extraction treatment | 68/236 (28.8) |
| Extraction with TAD anchorage | 112/236 (47.5) |
| Extraction with Class II elastics | 56/236 (23.7) |
| Two maxillary TADs | 94/112 (83.9) |
| Four TADs, two maxillary and two mandibular | 18/112 (16.1) |
| Total TADs placed | 260 |
| Mean devices per TAD patient | 2.32 ± 0.77 |
| Good elastic adherence, ≥ 20 hours per day | 47/56 (83.9) |
| Intermediate elastic adherence | 6/56 (10.7) |
| Poor elastic adherence | 3/56 (5.4) |

*Panel B. Mandibular retention regimen. Every patient received a maxillary vacuum-formed retainer.*

| **Regimen** | **Overall cohort, n (%)** | **With 12-month record, n (%)** |
| --- | --- | --- |
| Bonded fixed retainer | 126/236 (53.4) | 120/126 (95.2) |
| Vacuum-formed retainer | 45/236 (19.1) | 43/45 (95.6) |
| Fixed plus vacuum-formed | 65/236 (27.5) | 61/65 (93.8) |
| **Total** | **236** | **224** |

*Panel C. Retainer adherence.*

| **Regimen** | **Full-time use, 0–6 months** | **Night-time use, 6–12 months** |
| --- | --- | --- |
| Fixed | 119/126 (94.4) | 111/120 (92.5) |
| Removable | 44/45 (97.8) | 41/43 (95.3) |
| Combined | 58/65 (89.2) | 55/61 (90.2) |
| **Overall** | **221/236 (93.6)** | **207/224 (92.4)** |

*TAD, temporary anchorage device. Miniscrews were self-drilling, 1.6 mm in diameter and 8 mm long, placed in the buccal alveolar bone between the maxillary second premolar and first molar. Adherence was recorded at each four-weekly visit from the patient diary and, for elastics, from consumption. Mechanics and retention regimen were assigned by written clinical indication and not at random; the counts above therefore describe the cohort and do not support causal comparison between regimens.*

**Supplementary Table S5. Baseline comparison of patients with and without a 12-month record, and accounting of missing data**

*Panel A. Baseline comparison.*

| **Baseline variable** | **T2 available (n = 224)** | **T2 unavailable (n = 12)** | **Standardised mean difference** |
| --- | --- | --- | --- |
| Age, years | 21.4 ± 3.1 | 21.8 ± 3.4 | 0.12 |
| Female, n (%) | 130/224 (58.0) | 6/12 (50.0) | 0.16 |
| Overjet, mm | 6.8 ± 1.1 | 6.9 ± 1.2 | 0.09 |
| Crowding, mm | 2.7 ± 0.9 | 2.8 ± 1.0 | 0.11 |
| ANB (°) | 7.35 ± 0.74 | 7.42 ± 0.78 | 0.09 |
| U1-NA (°) | 32.26 ± 3.25 | 32.70 ± 3.40 | 0.13 |
| UL–E line, mm | 2.60 ± 0.26 | 2.63 ± 0.29 | 0.11 |
| Extraction treatment, n (%) | 159/224 (71.0) | 9/12 (75.0) | 0.09 |
| TAD anchorage, n (%) | 106/224 (47.3) | 6/12 (50.0) | 0.05 |
| Treatment duration, months | 21.3 ± 4.2 | 21.8 ± 4.5 | 0.12 |

*Panel B. Accounting of the cohort.*

| **Follow-up status** | **n (%)** |
| --- | --- |
| Original cohort | 236 (100) |
| Completed T1 (immediate post-treatment) assessment | 236 (100) |
| Completed 12-month (T2) lateral cephalogram | 224 (94.9) |
| Lost to follow-up | 8 (3.4) |
| Incomplete 12-month record set | 4 (1.7) |
| Included in the 12-month stability analysis | 224 (94.9) |
| All 15 variables measurable among those included | 224/224 (100) |
| Values imputed | 0 |

*Standardised mean differences are reported in preference to P values, which are underpowered with 12 patients in one group. No baseline variable reached a standardised mean difference of 0.20, so there is no observable imbalance between patients with and without a 12-month record. Selection bias arising from unmeasured differences cannot be excluded. All fifteen variables were measurable on the 12-month cephalogram in every one of the 224 patients retained; no value was imputed and no variable-specific exclusion occurred.*

**Supplementary Table S6. Full hard–soft tissue correlation matrix at T1 and T2**

| **Hard tissue** | **Soft tissue** | **T1 r** | **T1 P** | **T2 r** | **T2 P** |
| --- | --- | --- | --- | --- | --- |
| ANB | Nasolabial angle | 0.122 | 0.061 | 0.118 | 0.074 |
| ANB | Mentolabial angle | −0.018 | 0.772 | −0.025 | 0.704 |
| **ANB** | **UL–E line** | **0.442** | **<0.001** | **0.418** | **<0.001** |
| **ANB** | **LL–E line** | **0.482** | **<0.001** | **0.455** | **<0.001** |
| ANB | Chin thickness | 0.083 | 0.202 | 0.076 | 0.244 |
| Y-axis | Nasolabial angle | 0.034 | 0.599 | 0.029 | 0.654 |
| Y-axis | Mentolabial angle | 0.049 | 0.450 | 0.055 | 0.402 |
| Y-axis | UL–E line | −0.039 | 0.543 | −0.041 | 0.529 |
| Y-axis | LL–E line | −0.065 | 0.318 | −0.071 | 0.276 |
| Y-axis | Chin thickness | −0.038 | 0.559 | −0.045 | 0.490 |
| **U1-NA** | **Nasolabial angle** | **−0.508** | **<0.001** | **−0.486** | **<0.001** |
| U1-NA | Mentolabial angle | 0.065 | 0.316 | 0.058 | 0.374 |
| U1-NA | UL–E line | −0.034 | 0.600 | −0.028 | 0.669 |
| U1-NA | LL–E line | −0.014 | 0.829 | −0.019 | 0.771 |
| U1-NA | Chin thickness | −0.063 | 0.331 | −0.069 | 0.291 |
| L1-NB | Nasolabial angle | −0.018 | 0.778 | −0.024 | 0.715 |
| L1-NB | Mentolabial angle | −0.058 | 0.370 | −0.052 | 0.427 |
| L1-NB | UL–E line | −0.068 | 0.294 | −0.060 | 0.358 |
| L1-NB | LL–E line | 0.012 | 0.850 | 0.018 | 0.782 |
| L1-NB | Chin thickness | 0.109 | 0.093 | 0.103 | 0.114 |
| **U1-L1** | **Nasolabial angle** | **0.464** | **<0.001** | **0.441** | **<0.001** |
| U1-L1 | Mentolabial angle | −0.006 | 0.919 | −0.012 | 0.854 |
| U1-L1 | UL–E line | −0.058 | 0.372 | −0.063 | 0.334 |
| U1-L1 | LL–E line | −0.022 | 0.728 | −0.030 | 0.646 |
| U1-L1 | Chin thickness | 0.111 | 0.088 | 0.105 | 0.108 |

*Bold rows are significant after Bonferroni correction (α = 0.002); n = 236 at T1 and 224 at T2. T1 coefficients were computed on the three-dimensional measurements and T2 coefficients on the conventional cephalogram. Pearson coefficients are dimensionless, and the same-scan agreement documented in Supplementary Table S3 was high, so the two sets of coefficients are compared descriptively and were not tested against one another. The Y-axis angle, the only proxy for the vertical skeletal pattern recorded in this cohort, shows no significant association with any soft-tissue variable at either time point.*

**Supplementary Table S7. Correlations between hard- and soft-tissue change scores**

| **Hard-tissue change** | **Soft-tissue change** | **r** | **P** |
| --- | --- | --- | --- |
| ΔU1-NA angle (T0–T1) | Δ nasolabial angle (T0–T1) | −0.52 | <0.001 |
| ΔU1-NA mm (T0–T1) | ΔUL–E line (T0–T1) | 0.56 | <0.001 |
| ΔL1-NB mm (T0–T1) | ΔLL–E line (T0–T1) | 0.39 | <0.001 |
| ΔANB (T0–T1) | ΔUL–E line (T0–T1) | 0.34 | <0.001 |
| ΔANB (T0–T1) | ΔLL–E line (T0–T1) | 0.37 | <0.001 |
| ΔU1-L1 angle (T0–T1) | Δ nasolabial angle (T0–T1) | 0.46 | <0.001 |
| ΔY-axis angle (T0–T1) | Δ soft-tissue variables | −0.05 to 0.08 | > 0.05 |
| ΔU1-NA mm (T1–T2) | ΔUL–E line (T1–T2) | 0.31 | <0.001 |
| ΔL1-NB mm (T1–T2) | ΔLL–E line (T1–T2) | 0.26 | 0.002 |
| ΔU1-NA angle (T1–T2) | Δ nasolabial angle (T1–T2) | −0.29 | <0.001 |

*Change scores from T0 to T1 were computed on the three-dimensional measurements; change scores from T1 to T2 were computed on the cephalometric measurements. The coefficients between r = 0.39 and r = 0.56 correspond to 15% to 31% of shared variance, so the greater part of the between-patient variation in soft-tissue change is not explained by the dentoalveolar change.*

**Supplementary Table S8. Complete multivariable regression models, model fit and diagnostics (n = 236)**

*Panel A. Full models. Every covariate is shown.*

| **Outcome** | **Predictor** | **B (95% CI)** | **β** | **P** | **VIF** |
| --- | --- | --- | --- | --- | --- |
| Δ Nasolabial angle | ΔU1-NA angle (°) | −0.96 (−1.20 to −0.72) | −0.36 | <0.001 | 1.44 |
|  | Age | +0.05 (−0.18 to +0.28) | +0.02 | 0.667 | 1.09 |
|  | Female sex | +0.62 (−1.05 to +2.29) | +0.04 | 0.465 | 1.06 |
|  | Baseline nasolabial angle | −0.29 (−0.40 to −0.18) | −0.33 | <0.001 | 1.18 |
|  | Extraction treatment | +2.08 (+0.54 to +3.62) | +0.12 | 0.008 | 1.71 |
|  | Treatment duration | +0.04 (−0.13 to +0.21) | +0.02 | 0.643 | 1.53 |
| Δ UL–E line | ΔU1-NA (mm) | +0.24 (+0.18 to +0.30) | +0.44 | <0.001 | 1.57 |
|  | Age | −0.001 (−0.009 to +0.007) | −0.01 | 0.801 | 1.08 |
|  | Female sex | −0.02 (−0.10 to +0.06) | −0.03 | 0.622 | 1.07 |
|  | Baseline UL–E line | −0.36 (−0.48 to −0.24) | −0.25 | <0.001 | 1.14 |
|  | Extraction treatment | −0.19 (−0.30 to −0.08) | −0.20 | 0.001 | 1.76 |
|  | Treatment duration | −0.004 (−0.012 to +0.004) | −0.04 | 0.326 | 1.58 |
| Δ LL–E line | ΔL1-NB (mm) | +0.16 (+0.10 to +0.22) | +0.31 | <0.001 | 1.48 |
|  | ΔANB (°) | +0.11 (+0.04 to +0.18) | +0.17 | 0.002 | 1.36 |
|  | Age | +0.001 (−0.007 to +0.009) | +0.01 | 0.809 | 1.08 |
|  | Female sex | −0.03 (−0.11 to +0.05) | −0.03 | 0.461 | 1.06 |
|  | Baseline LL–E line | −0.29 (−0.42 to −0.16) | −0.22 | <0.001 | 1.17 |
|  | Extraction treatment | −0.12 (−0.23 to −0.01) | −0.13 | 0.033 | 1.68 |
|  | Treatment duration | −0.002 (−0.010 to +0.006) | −0.02 | 0.623 | 1.55 |
| Δ Mentolabial angle | ΔL1-NB angle (°) | −0.54 (−0.86 to −0.22) | −0.19 | 0.001 | 1.42 |
|  | Age | +0.08 (−0.16 to +0.32) | +0.04 | 0.512 | 1.09 |
|  | Female sex | +0.74 (−0.95 to +2.43) | +0.05 | 0.389 | 1.06 |
|  | Baseline mentolabial angle | −0.27 (−0.38 to −0.16) | −0.31 | <0.001 | 1.15 |
|  | Extraction treatment | +1.32 (−0.28 to +2.92) | +0.09 | 0.105 | 1.70 |
|  | Treatment duration | +0.06 (−0.11 to +0.23) | +0.04 | 0.487 | 1.54 |
| Δ Chin thickness | ΔL1-NB angle (°) | −0.015 (−0.040 to +0.010) | −0.07 | 0.238 | 1.42 |
|  | Age | +0.006 (−0.004 to +0.016) | +0.03 | 0.238 | 1.09 |
|  | Female sex | +0.05 (−0.02 to +0.12) | +0.03 | 0.161 | 1.06 |
|  | Baseline chin thickness | −0.31 (−0.39 to −0.23) | −0.46 | <0.001 | 1.20 |
|  | Extraction treatment | +0.17 (−0.02 to +0.36) | +0.10 | 0.078 | 1.69 |
|  | Treatment duration | +0.004 (−0.003 to +0.011) | +0.02 | 0.261 | 1.53 |

*Panel B. Model fit.*

| **Outcome** | **R²** | **Adjusted R²** | **Overall model** |
| --- | --- | --- | --- |
| Δ Nasolabial angle | 0.356 | 0.339 | F(6, 229) = 21.10; P < 0.001 |
| Δ UL–E line | 0.412 | 0.397 | F(6, 229) = 26.74; P < 0.001 |
| Δ LL–E line | 0.278 | 0.256 | F(7, 228) = 12.54; P < 0.001 |
| Δ Mentolabial angle | 0.168 | 0.146 | F(6, 229) = 7.71; P < 0.001 |
| Δ Chin thickness | 0.231 | 0.211 | F(6, 229) = 11.46; P < 0.001 |

*Panel C. Residual and influence diagnostics.*

| **Outcome** | **Durbin–Watson** | **Shapiro–Wilk P** | **Breusch–Pagan P** | **Maximum Cook's D** | **Maximum leverage** |
| --- | --- | --- | --- | --- | --- |
| Δ Nasolabial angle | 1.96 | 0.124 | 0.318 | 0.012 | 0.054 |
| Δ UL–E line | 2.04 | 0.087 | 0.241 | 0.014 | 0.048 |
| Δ LL–E line | 1.91 | 0.162 | 0.416 | 0.015 | 0.059 |
| Δ Mentolabial angle | 2.07 | 0.073 | 0.287 | 0.011 | 0.051 |
| Δ Chin thickness | 1.98 | 0.109 | 0.352 | 0.013 | 0.056 |

*B is the unstandardised coefficient and β the standardised coefficient; VIF is the variance inflation factor. Anchorage is not entered in these models: every TAD patient and every elastics patient was an extraction patient, so extraction status is the exact sum of the two anchorage indicators and entering all three would produce perfect linear dependence. Anchorage is examined separately, within the 168 extraction patients, in Supplementary Table S11. The maximum VIF across all five models is 1.76, so no predictor is materially collinear; a threshold of 5 was adopted while recognising that such thresholds are conventions rather than tests. Cook's distance was assessed against a 4/n threshold (0.017); the largest observed value is 0.015, so no observation is influential. Residual-versus-fitted and normal probability plots were inspected for every model and showed no departure from linearity, homoscedasticity or normality. Because treatment was allocated by clinical indication, the extraction coefficient combines the effect of the mechanics with the effect of the indication for them; no attempt has been made to quantify residual confounding.*

**Supplementary Table S9. Extraction versus non-extraction subgroup analysis**

| **Variable** | **Extraction (n = 168)** | **Non-extraction (n = 68)** | **P** |
| --- | --- | --- | --- |
| ΔU1-NA (°), T0–T1 | −7.1 ± 2.8 | −3.9 ± 2.1 | <0.001 |
| ΔU1-NA (mm), T0–T1 | −3.7 ± 0.6 | −2.1 ± 0.5 | <0.001 |
| ΔL1-NB (°), T0–T1 | −5.0 ± 2.5 | −3.3 ± 2.2 | <0.001 |
| Δ Nasolabial angle (°), T0–T1 | +11.1 ± 7.8 | +5.9 ± 6.4 | <0.001 |
| ΔUL–E line (mm), T0–T1 | −2.28 ± 0.32 | −1.58 ± 0.29 | <0.001 |
| ΔLL–E line (mm), T0–T1 | −1.25 ± 0.30 | −0.86 ± 0.26 | <0.001 |
| ΔUL–E line (mm), T1–T2 | +0.09 ± 0.18 | +0.05 ± 0.15 | 0.110 |
| Δ Nasolabial angle (°), T1–T2 | −0.62 ± 2.4 | −0.38 ± 2.1 | 0.480 |

*Mean change ± SD; between-group comparison by independent t test. T0–T1 changes are three-dimensional; T1–T2 changes are cephalometric. The extraction group showed larger immediate dentoalveolar and soft-tissue change, and neither T1–T2 change reached the combined SDD of its variable in either group (Supplementary Table S2). These are group-associated differences and not treatment effects: extraction was chosen for the patients with the larger overjet, greater crowding and more convex profile, so the groups differed at baseline by construction.*

**Supplementary Table S10. TAD versus Class II elastic anchorage among extraction cases**

| **Variable** | **TAD (n = 112)** | **Class II elastics (n = 56)** | **P** |
| --- | --- | --- | --- |
| ΔU1-NA (°), T0–T1 | −7.5 ± 2.7 | −6.3 ± 2.8 | 0.012 |
| ΔU1-NA (mm), T0–T1 | −3.9 ± 0.6 | −3.4 ± 0.6 | <0.001 |
| ΔL1-NB (°), T0–T1 | −4.6 ± 2.4 | −5.7 ± 2.6 | 0.009 |
| ΔANB (°), T0–T1 | −2.1 ± 0.7 | −1.8 ± 0.6 | 0.018 |
| Δ Nasolabial angle (°), T0–T1 | +11.8 ± 7.7 | +9.7 ± 7.5 | 0.095 |
| ΔUL–E line (mm), T0–T1 | −2.35 ± 0.31 | −2.12 ± 0.30 | <0.001 |
| ΔLL–E line (mm), T0–T1 | −1.18 ± 0.29 | −1.38 ± 0.31 | <0.001 |
| ΔUL–E line (mm), T1–T2 | +0.09 ± 0.19 | +0.07 ± 0.17 | 0.512 |
| Δ Nasolabial angle (°), T1–T2 | −0.58 ± 2.0 | −0.49 ± 1.9 | 0.771 |

*Mean change ± SD; between-group comparison by independent t test. Changes differed according to anchorage category, maxillary incisor and upper-lip change being greater with skeletal anchorage and mandibular incisor and lower-lip change greater with intermaxillary elastics. Neither T1–T2 change reached the combined SDD of its variable in either group. The qualitative descriptors of relapse used in an earlier version of this table have been withdrawn and replaced by the mean change with its standard deviation. Anchorage was selected clinically, so these are group-associated differences and not causal effects of the anchorage system.*

**Supplementary Table S11. Anchorage sensitivity models restricted to extraction patients (n = 168)**

| **Outcome** | **Main hard-tissue coefficient, B (95% CI)** | **TAD versus elastics, B (95% CI)** | **P for anchorage** |
| --- | --- | --- | --- |
| Δ Nasolabial angle | −0.90 (−1.17 to −0.63) | +0.84 (−0.55 to +2.23) | 0.235 |
| Δ UL–E line | +0.22 (+0.15 to +0.29) | −0.10 (−0.19 to −0.01) | 0.031 |
| Δ LL–E line | +0.15 (+0.08 to +0.22) | +0.12 (+0.03 to +0.21) | 0.010 |
| Δ Mentolabial angle | −0.50 (−0.87 to −0.13) | +0.72 (−0.81 to +2.25) | 0.354 |
| Δ Chin thickness | −0.01 (−0.04 to +0.02) | +0.06 (−0.11 to +0.23) | 0.486 |

*Models are otherwise identical to those in Supplementary Table S8 and are adjusted for age, sex, the baseline value of the outcome and treatment duration. Restricting the analysis to extraction patients removes the collinearity between extraction status and the anchorage indicators and permits a valid comparison of the two anchorage systems. Anchorage category was associated with upper- and lower-lip position but not with the angular soft-tissue outcomes. Because the anchorage system was selected on clinical grounds, these coefficients describe association and not causation.*

**Supplementary Table S12. Retention regimen: stability and coefficient sensitivity**

*Panel A. T1–T2 change by retainer regimen (mean ± SD).*

| **T1–T2 outcome** | **Fixed (n = 120)** | **Removable (n = 43)** | **Combined (n = 61)** | **Omnibus P** |
| --- | --- | --- | --- | --- |
| U1-NA change (°) | +0.39 ± 0.93 | +0.31 ± 0.88 | +0.36 ± 0.96 | 0.841 |
| L1-NB change (°) | +0.25 ± 0.85 | +0.19 ± 0.79 | +0.23 ± 0.87 | 0.903 |
| Nasolabial-angle change (°) | −0.57 ± 1.98 | −0.49 ± 1.84 | −0.55 ± 2.04 | 0.934 |
| UL–E change (mm) | +0.08 ± 0.20 | +0.07 ± 0.18 | +0.09 ± 0.21 | 0.862 |
| LL–E change (mm) | +0.06 ± 0.17 | +0.05 ± 0.16 | +0.06 ± 0.18 | 0.941 |

*Panel B. Stability of the principal coefficients when retainer regimen is added to the model.*

| **Principal association** | **Original B** | **With retainer regimen** | **Relative change** |
| --- | --- | --- | --- |
| ΔU1-NA angle → Δ nasolabial angle | −0.96 | −0.94 | 2.1% |
| ΔU1-NA mm → ΔUL–E line | +0.24 | +0.23 | 4.2% |
| ΔL1-NB mm → ΔLL–E line | +0.16 | +0.16 | 0.0% |
| ΔL1-NB angle → Δ mentolabial angle | −0.54 | −0.52 | 3.7% |
| Extraction → Δ chin thickness | +0.17 | +0.16 | 5.9% |

*Omnibus P values are from one-way analysis of variance across the three regimens. No coefficient changed by more than 5.9% when retainer regimen was added, supporting the statement that the principal associations are not driven by the retention protocol. These comparisons must not be read as evidence that the regimens are equivalent: retainer type was assigned by written indication, so the groups differ in their original relapse risk, and the analysis is a check on the coefficients of interest rather than an estimate of a retention effect.*

**Supplementary figure legends**

**Supplementary Figure S1.** Post-treatment scatter plots of ANB against each soft-tissue variable (n = 236). Measurements are from the three-dimensional reconstruction at T1.

**Supplementary Figure S2.** Post-treatment scatter plots of the Y-axis angle against each soft-tissue variable (n = 236). Measurements are from the three-dimensional reconstruction at T1.

**Supplementary Figure S3.** Post-treatment scatter plots of U1-NA against each soft-tissue variable (n = 236). Measurements are from the three-dimensional reconstruction at T1.

**Supplementary Figure S4.** Post-treatment scatter plots of L1-NB against each soft-tissue variable (n = 236). Measurements are from the three-dimensional reconstruction at T1.

**Supplementary Figure S5.** Post-treatment scatter plots of U1-L1 against each soft-tissue variable (n = 236). Measurements are from the three-dimensional reconstruction at T1.
